# Structural Brain Correlates Associated with Professional Handball Playing

**DOI:** 10.1371/journal.pone.0124222

**Published:** 2015-04-27

**Authors:** Jürgen Hänggi, Nicolas Langer, Kai Lutz, Karin Birrer, Susan Mérillat, Lutz Jäncke

**Affiliations:** 1 Division Neuropsychology, Department of Psychology, University of Zurich, Zurich, Switzerland; 2 Neural Systems Lab, The City College of New York, New York, NY, United States of America; 3 Child Mind Institute, New York, NY, United States of America; 4 Center for Neurology and Rehabilitation, cereneo AG, Vitznau, Switzerland; 5 Department of Neurology, University Hospital Zurich, Zurich, Switzerland; 6 Rehabilitation Center Affoltern am Albis, University Children’s Hospital Zurich, Affoltern am Albis, Switzerland; 7 International Normal Aging and Plasticity Imaging Center (INAPIC), University of Zurich, Zurich, Switzerland; 8 Center for Integrative Human Physiology (ZIHP), University of Zurich, Zurich, Switzerland; 9 University Research Priority Program (URPP), Dynamic of Healthy Aging, University of Zurich, Zurich, Switzerland; 10 Department of Special Education, King Abdulaziz University, Jeddah, Saudi Arabia; Beijing Normal University,Beijing, CHINA

## Abstract

**Background:**

There is no doubt that good bimanual performance is very important for skilled handball playing. The control of the non-dominant hand is especially demanding since efficient catching and throwing needs both hands.

**Methodology/Hypotheses:**

We investigated training-induced structural neuroplasticity in professional handball players using several structural neuroimaging techniques and analytic approaches and also provide a review of the literature about sport-induced structural neuroplastic alterations. Structural brain adaptations were expected in regions relevant for motor and somatosensory processing such as the grey matter (GM) of the primary/secondary motor (MI/supplementary motor area, SMA) and somatosensory cortex (SI/SII), basal ganglia, thalamus, and cerebellum and in the white matter (WM) of the corticospinal tract (CST) and corpus callosum, stronger in brain regions controlling the non-dominant left hand.

**Results:**

Increased GM volume in handball players compared with control subjects were found in the right MI/SI, bilateral SMA/cingulate motor area, and left intraparietal sulcus. Fractional anisotropy (FA) and axial diffusivity were increased within the right CST in handball players compared with control women. Age of handball training commencement correlated inversely with GM volume in the right and left MI/SI and years of handball training experience correlated inversely with radial diffusivity in the right CST. Subcortical structures tended to be larger in handball players. The anatomical measures of the brain regions associated with handball playing were positively correlated in handball players, but not interrelated in control women.

**Discussion/Conclusion:**

Training-induced structural alterations were found in the somatosensory-motor network of handball players, more pronounced in the right hemisphere controlling the non-dominant left hand. Correlations between handball training-related measures and anatomical differences suggest neuroplastic adaptations rather than a genetic predisposition for a ball playing affinity. Investigations of neuroplasticity specifically in sportsmen might help to understand the neural mechanisms of expertise in general.

## Introduction

Neuroplasticity, the capacity of the central nervous system to modify its structural and functional organization, involves highly complex, multistep processes that include numerous time-dependent (and maybe less time-dependent) events that occur at different levels such as the molecular, synaptic, cellular, electrophysiological, and structural organization level [1]. The scope of neuroplasticity is rather wide ranging from short-term weakening and strengthening of existing synapses through induction of long-term potentiation or depression, to the formation of long-lasting new neuronal connections (synaptogenesis), the formation of new glial (gliogenesis) or even neuronal cells (neurogenesis), the formation of new blood vessels (angiogenesis) [1,2], and other potential, but here unmentioned mechanisms as well.

The sum of the above-mentioned microscopic and mesoscopic brain alterations, whether functional or structural in nature, can be measured in vivo by using a variety of magnetic resonance imaging (MRI) techniques [3]. T1-weighted and diffusion-weighted pulse sequences combined with computational neuromorphometric procedures have shown to be a valuable methodology for the investigation of structural brain differences between groups or alterations over time in the diseased as well as in the healthy brain [4–8]. Among the first longitudinally studies that investigated practice-dependent structural neuroplasticity by using MRI were the meanwhile famous juggling studies conducted by Draganski and colleagues [9–12]. These studies suggest that even short-term practice (weeks to months) of a specific task (here a visuospatial-motor task, i.e. juggling) is associated with structural adaptations in relevant brain regions that can be transient or permanent. In addition, these longitudinal studies suggest also that the alterations reported are the consequences of the training and hence evoked by neuroplastic processes and not just the result of a genetic predisposition for a particular neural trait. Therefore, it is conceivable to assume that long-term training (years to decades) in a specific task evokes structural brain adaptations too and that those adaptations can be measured using structural MRI in cross-sectional study designs.

Indeed, there is strong evidence from cross-sectional structural MRI studies that sensory, motor, and cognitive training modulates brain morphology [3,8,13–15]. In the field of sport, there is a small corpus of structural neuroimaging studies (see below) using T1-weighted as well as diffusion-weighted MR pulse sequences combined with neuromorphometric procedures that revealed different morphological alterations in grey matter (GM) and white matter (WM) of sportsmen compared with laymen.

In two previous studies of our group, the morphometric features of brain areas relevant for motor and sensory control were investigated in professional female ballet dancers [16] and in professional male golf players [17]. These studies revealed alterations in the GM and WM architecture of the somatosensory-motor network including the premotor cortex, supplementary motor area (SMA), parietal cortex, putamen, corticospinal tract (CST), internal capsule, corpus callosum, and anterior cingulum. Increases in GM volumes due to golf training were also found in the primary motor (MI) and premotor cortex in a longitudinal study in healthy elderly subjects who started playing golf as a leisure activity [18]. A comprehensive review of the literature about structural brain differences in amateur and professional sportsmen compared with laymen is provided in Table 1.

**Table 1:** A comprehensive review of the literature about structural brain differences in sportsmen compared with laymen.

					Brain regions with morphometric differences													
Study	Sport	# Exp. Con.	# Fem. Male	Imaging modality / Methods	MI	SI	SMA	CMA	PMC	SII	BG	Thal.	Cer.	CST	IC	EC	CC	Cin.
Present study	Handball players	11 12	11 / 0 12 / 0	VBM / SBM / DTI	GM ↑	GM ↑	GM ↑	GM ↑	GM ↑	GM↑	GM↑	GM ↑	GM↑WM ↓	FA ↓	-	-	-	-
Hänggi et al., 2010	Ballet dancers	10 10	10 / 0 10 / 0	VBM / DTI	-	-	GM ↓	-	GM↓FA ↓	-	GM↓	-	-	WM ↓	WM ↓	-	WM↓	WM↓
Jäncke et al., 2009	Golf players	20 20	0 / 20 0 / 20	VBM / DTI	-	-	-	-	GM ↑	-	WM↓	-	-	FA↓WM ↓	FA↓WM ↓	WM↓	WM↓	-
Park et al., 2006	Basketball players	19 20	0 / 19 0 / 20	Manual volumetry	n.a.	n.a.	n.a.	n.a.	n.a.	n.a.	n.a.	n.a.	-	n.a.	n.a.	n.a.	n.a.	n.a.
Park et al., 2009	Basketball players	19 20	0 / 19 0 / 20	Manual volumetry	n.a.	n.a.	n.a.	n.a.	n.a.	n.a.	n.a.	n.a.	GM↑WM ↑	n.a.	n.a.	n.a.	n.a.	n.a.
Park et al., 2011	Basketball players	19 20	0 / 19 0 / 20	Manual volumetry	n.a.	n.a.	n.a.	n.a.	n.a.	n.a.	GM↑	n.a.	n.a.	n.a.	n.a.	n.a.	n.a.	n.a.
Park et al., 2012	Speed skaters	16 18	0 / 16 0 / 18	Manual volumetry	n.a.	n.a.	n.a.	n.a.	n.a.	n.a.	n.a.	n.a.	GM↑WM ↑	n.a.	n.a.	n.a.	n.a.	n.a.
Wei et al., 2009	High diving players	12 12	6 / 6 6 / 6	VBM	-	-	-	-	GM ↑	-	GM↓	GM ↓	GM ↓	-	-	-	-	-
Wei et al., 2011	High diving players	12 12	6 / 6 6 / 6	SBM	-	-	-	-	-	-	n.a.	n.a.	n.a.	n.a.	n.a.	n.a.	n.a.	n.a.
Zhang et al., 2013	High diving players	12 12	6 / 6 6 / 6	Sub-cortical	n.a.	n.a.	n.a.	n.a.	n.a.	n.a.	GM↑	GM ↑	n.a.	n.a.	n.a.	n.a.	n.a.	n.a.
Di et al., 2012	Badminton players	20 18	10 / 10 9 / 9	VBM	-	-	-	-	-	-	-	-	GM ↑	n.a.	n.a.	n.a.	n.a.	n.a.
Zhang et al., 2012	Mounte-neers	14 0	6 / 8 0 / 0	DTI°	-	-	-	-	-	-	-	-	-	FA ↓	FA ↓	-	FA↓	FA↓
Jacini et al., 2009	Judo wrestlers	8 18	0 / 8 0 / 18	VBM	GM ↑	GM ↑	GM ↑	-	-	-	-	-	-	n.a.	n.a.	n.a.	n.a.	n.a.
Hüfner et al., 2010	Dancers* / slackliners	21 20	11 / 10 12 / 8	VBM	-	-	-	-	-	-	-	GM ↓	GM ↓	-	-	-	-	GM↑
Wang et al., 2013	World-class gymnasts	13 14	7 / 6 7 / 7	DTI	LE ↑	LE ↑	-	-	-	-	-	LE ↓	-	FA ↓	-	-	-	LE↓
Huang et al., 2013	World-class gymnasts	13 14	7 / 6 7 / 7	VBM / DTI	GM↑FA ↓	GM↑FA ↓	FA ↓	-	FA ↓	-	-	-	-	-	-	-	-	FA↓
Di Paola et al., 2013	Climbers	10 10	0 / 10 0 / 10	VBM	n.a.	n.a.	n.a.	n.a.	n.a.	n.a.	n.a.	n.a.	GM ↓	n.a.	n.a.	n.a.	n.a.	n.a.
Schlaffke et al., 2014	Martial artists	1313	0 /13 0 / 13	VBM	-	-	GM ↑	-	GM ↑	-	-	-	-	n.a.	n.a.	n.a.	n.a.	n.a.
Schlaffke et al., 2014	Endurance athletes	13 13	0 /13 0 / 13	VBM	-	-	GM ↑	-	GM ↑	-	-	-	-	n.a.	n.a.	n.a.	n.a.	n.a.

*, ballet and ice dancers;

^°^, longitudinal study;

^↑^, increase;

^↓^, decrease;

CC, corpus callosum; Cer, cerebellum; Cin., cingulum; CMA, cingulate motor area; Con, controls; CST, corticospinal tract; DTI, diffusion tensor imaging; EC, external capsule; FA, fractional anisotropy; Fem., female; GM, grey matter; IC, internal capsule; LE, local network efficiency; MI, primary motor cortex; n.a., not assessed; PMC, premotor cortex; SBM, surface-based morphometry; SI, primary somatosensory cortex; SII, secondary somatosensory cortex; SMA, supplementary motor area; Thal., thalamus; VBM, voxel-based morphometry; WM, white matter.

The reviewed studies based on T1- and diffusion-weighted MRI data combined with voxel-based (VBM) or surface-based morphometry (SBM), manual volumetry (tracing), voxel-wise analyses of diffusion parameters, or fibre tractography combined with graph-theoretical (network) analyses. In summary, the review shows that local GM and WM morphology is altered in sportsmen compared with laymen in the somatosensory-motor network including the MI and primary and secondary somatosensory cortex (SI, SII), premotor cortex, SMA, basal ganglia, thalamus, cerebellum, CST, and corpus callosum [16,17,19–33].

Although it is well established that finger/hand/arm performance (a.k.a. handedness) is related to motor cortex morphology [34], there is no study so far that focuses explicitly on brain plasticity related to bimanual and non-dominant hand demands in a sport. Due to the fact that the MI and SI hand area are overrepresented in the human brain, training-induced, neuroplastic structural alterations might be more easily detected if such large areas are involved. Indeed, there is evidence for structural adaptations in the MI and SI hand representation in the musician’s (e.g., pianist’s) brain, another expert group demanded by non-dominant and bimanual finger and hand movements [35,36].

Although there is preliminary evidence for structural alterations in the hand representations of judo wrestlers [23] and world-class gymnasts [21,28], it remains unclear to what extent the hand movements of wrestlers and gymnasts differ from those movements used in everyday life. This is important because influences on the hand representations evoked by hand movements used in everyday life are also present in the control subjects (non-sportsmen) commonly used in such investigations. However, there is no doubt that good bimanual performance is essential for skilled handball playing. The control of the non-dominant hand is especially demanding since efficient catching and throwing needs both hands.

In the present study, we investigated structural brain alterations associated with long-term, intensive and extensive handball training and experience and focused on the motor and somatosensory aspect of handball playing, although efficient processing of visuospatial information and its transformations into executive motor programs are also vital for excellent handball playing. Long-term structural brain adaptations were expected to be evident in the somatosensory-motor network including MI, SMA, premotor cortex, SI, SII, basal ganglia, thalamus, and cerebellum in the case of GM and in the CST and corpus callosum in the case of WM.

## Materials and Methods

### Ethics statement

The research reported in the present study was conducted according to the principles expressed in the Declaration of Helsinki. The local ethics committee of the canton Zurich, Switzerland, approved the study and it’s protocols and written informed consent was obtained from all participants prior to study enrolment. The subjects were paid for participation in the study.

### Subjects

Twenty-five women with a mean age of 24.4 years (standard deviation, SD = 3.06 years) participated in the study. Thirteen participants were professional handball players, who played in the highest national league and some of them were members of the Swiss national female handball team, and 12 were control women matched with respect to handedness and age, who never trained handball or any related ball game such as basketball or volleyball. Except for the regular flute lessons at school, none of the handball players and control women had a formal musical education or learnt to play any musical instrument at an advanced level. Two handball players had to be excluded; one due to a loss of the diffusion-weighted as well as functional brain data (the latter of which however are not part of the present study) and one due to a severe imaging artefact in her T1-weighted scan. Hence, the group of handball players consisted of 11 players with a mean age of 23.6 years (SD = 2.9 years), who were compared against 12 control women with a mean age of 25.5 years (SD = 2.9 years) who were matched with respect to handedness and age. All participants were consistent right-handers as assessed by the hand dominance test [37,38]. Participants had no history of neurologic or psychiatric disorders, showed no neuropsychological problems, and denied taking drugs or any illegal medication.

### Magnetic resonance imaging data acquisition

MRI scans were acquired on a 3.0 Tesla Philips Intera whole body scanner (Philips Medical Systems, Best, The Netherlands) equipped with a transmit-receive body coil and a commercial eight-element head coil array that is capable of sensitivity encoding (SENSE).

A volumetric 3D T1-weighted gradient echo sequence (turbo field echo) image was measured with a spatial resolution of 1 × 1 × 1.5 mm^3^ (acquisition matrix 224 × 224 pixels, 90 slices) and reconstructed to a resolution of 0.86 × 0.86 × 0.75 mm^3^ (reconstructed matrix 256 × 256 pixels, 180 slices). Further imaging parameters were: Field of view (FOV) = 220 × 220 mm^2^, echo-time (TE) = 2.3 ms, repetition-time (TR) = 20 ms, flip-angle α = 20°, SENSE factor R = 2.0. Total acquisition time was about 4.24 minutes.

A diffusion-weighted spin echo, echo-planar imaging sequence was used to obtain diffusion-weighted scans with a measured spatial resolution of 2.29 × 2.34 × 2.50 mm^3^ (acquisition matrix 96 × 94 pixels, 55 slices) and a reconstructed spatial resolution of 1.72 × 1.72 × 2.50 mm^3^ (reconstruction matrix 128 x 128 pixels, 55 slices). Further imaging parameters were: FOV = 220 x 220 mm^2^; TE = 50.0 ms; TR = 11,300 ms; α = 90°; SENSE factor R = 2.1; b-value b = 1,000 s/mm^2^; and number of averages = 2. Diffusion was measured in 15 non-collinear directions preceded by a non-diffusion-weighted volume (reference volume). Total acquisition time was about 17.7 minutes.

### Voxel-based morphometry

Between-group differences in GM volume were evaluated by using VBM [39,40]. T1-weighted MRI scans were preprocessed and analysed with the FSL-VBM tool [41] (http://fsl.fmrib.ox.ac.uk/fsl/fslwiki/FSLVBM), an optimised VBM protocol [42] that is implemented in the FMRIB software library (FSL) version 5.0.1 [43] (www.fmrib.ox.ac.uk/fsl). First, structural images were brain-extracted and GM-segmented before being registered to the Montreal neurological institute (MNI) 152 standard space using non-linear registration [44]. The resulting images were averaged and flipped along the x-axis to create a left-right symmetric, study-specific GM template. Second, all native GM images were non-linearly registered to this study-specific template and "modulated" to correct for local expansion (inflation) and contraction (deflation) due to the non-linear component of the spatial transformation. The modulated GM images were then smoothed with different smoothing kernels (sigma 2, 3, 4 and 5) in order to determine, according to the matched filter theorem, the size of the smoothing kernel that is most sensitive for detecting effects of a particular size. This smoothing procedure, which is recommended by the FSL-VBM user guide (see part E1 and E2 under http://fsl.fmrib.ox.ac.uk/fsl/fslwiki/FSLVBM/UserGuide) revealed that the effects of the VBM group comparison (n = 23) are most sensitive with sigma = 5, whereas the effects of the VBM correlation (n = 11) are most sensitive with sigma = 3. We therefore decided to apply an isotropic Gaussian kernel with sigma = 5 (corresponding to 11.5 mm FWHM) for the group analysis and a sigma = 3 (corresponding to 6.9 mm FWHM) for the correlational analysis. It is important to note that this procedure, which however might be regarded by others as an issue of “cherry picking”, is scientifically valid and in accordance with the theory behind smoothing. These maps were then subjected to statistical analyses (see below).

To restrict the statistical analysis to the cortical regions of interest (ROIs) that are part of the somatosensory-motor network, we used probability maps derived from the Juelich histological atlas (https://www.jubrain.fz-juelich.de/apps/cytoviewer/cytoviewer-main.php) or from the Harvard-Oxford cortical and subcortical structural atlas (http://www.cma.mgh.harvard.edu/fsl_atlas.html) both implemented in FSL. Following probability maps were used from the Juelich histological atlas: Primary motor cortex, MI (Brodmann area, BA4a and BA4p) [45], primary somatosensory cortex, SI (BA1, BA2, BA3a, BA3b) [46], premotor cortex (BA6) [47]. The SMA was taken from the Harvard-Oxford cortical atlas [48–51] because this ROI is not available in the Juelich histological atlas. We used the anterior part of the cingulate gyrus (taken from the Harvard-Oxford cortical atlas) and divided it into an anterior and middle part and this middle part (MNI coordinates y = 32 to y = -8) was added to the mask to be able investigating potential brain differences also in the cingulate motor area (CMA), which is located ventrally to the SMA. The subcortical structures thalamus, caudate nucleus, putamen, and pallidum (taken from the Harvard-Oxford subcortical structural atlas) were additionally included. All these probability maps were combined into a single mask without thresholding. The thalamus, caudate nucleus, putamen, and pallidum have been investigated additionally as a whole (global effect) based on volumetric subcortical segmentations derived from FreeSurfer’s volumetric processing stream (see below).

As a negative control condition, we also investigated a brain area for which we did not expect any difference between groups. The primary and secondary visual cortex (BA17 and BA18) served as control regions and its probability masks [52] have been derived from the Juelich histological atlas implemented in FSL. These four probability maps were combined into a single mask without thresholding.

### Diffusion tensor imaging

Diffusion tensor imaging (DTI) data pre-processing and tract-based spatial statistical (TBSS) analysis were performed with FSL version 5.0.3 [43] (www.fmrib.ox.ac.uk/fsl). In a first step, non-brain tissue was automatically removed [53]. Further automated pre-processing steps (eddy-current and head movement correction) and the construction of individual diffusion tensor maps as well as FA and mean, axial, and radial diffusivity maps were performed using FMRIB’s diffusion toolbox 3.0 [43]. We then applied the tract-based spatial statistics (TBSS) tool [54] implemented in FSL. Linear and non-linear spatial registrations of the FA map into a standard stereo-tactic space (MNI space represented by the FMRIB58-FA template) were applied using FSL’s registration tools FLIRT and FNIRT, respectively. These transformations were then applied to the mean, axial and radial diffusivity maps using the TBSS_NON_FA tool. Next, the mean of all subjects’ aligned FA images was created, and then ‘thinned’ using standard image processing techniques to create a mean FA skeleton that represents the centres of major tracts common to the group of subjects. Each subject’s aligned FA data was then projected (perpendicular to the local tract direction) onto this skeleton so that the projected FA values are taken from the centres of the tracts in the original FA image [55]. These maps were then subjected to statistical analyses (see below).

Axial and radial diffusivity are measures of the diffusion magnitude parallel and perpendicular to the axons, respectively, within microstructural WM compartments. Here, these two parameters were chosen as measures of interest besides of commonly used FA and mean diffusivity, which rather provide unspecific diffusion information. Axial diffusivity is represented by the first eigenvalue, whereas radial diffusivity was computed as mean of the second and third eigenvalue.

To restrict the statistical analysis to the fibres of the CST and corpus callosum within the skeletonized maps (see above), we used the probability maps of the left and right CST and that of the corpus callosum derived from the Juelich histological atlas [56,57] implemented in FSL. The three probability maps were combined into a single mask without thresholding.

As a negative control condition, we also investigated a tract for which we did not expect any difference between groups. The cingulum served as a control tract and its probability masks (including the cingulate and hippocampal part) have been derived from the JHU (John Hopkins University) WM tractography atlas [58] implemented in FSL. These four probability maps were combined into a single mask without thresholding.

### Subcortical volumetric segmentations

The FreeSurfer image analysis suite (see above) has also a fully automated subcortical segmentation procedure that provides, with high accuracy, the volumes of the subcortical structures such as the basal ganglia, thalamus, cerebellum, and corpus callosum. The volumetric segmentation procedure is described in more detail elsewhere [59–61]. We investigated the volumes of the following structures: putamen, caudate nucleus, pallidum, thalamus, corpus callosum (divided into five anterior-posterior segments), and the cerebellum (divided into left and right GM and WM). For the corpus callosum, two different types of tissue measures are reported. Diffusivity measures derived from the DTI analysis (TBSS) as well as volumetric measures derived from the T1-weighted MRI-based segmentation procedure implemented in the FreeSurfer software.

### Surface-based morphometry

Surface-based morphometry (SBM) has been applied in addition to the VBM analysis for two reasons: First, we applied SBM to replicate the volumetric findings of the VBM approach by using a surface-based morphometric data pre-processing stream that is different from the pre-processing stream applied in the common VBM approach. Second, SBM was used to track down whether differences in volume (either revealed by the VBM or the SBM approach) are driven by differences in cortical thickness or cortical surface area. The full procedure applied in the SBM analysis of cortical thickness, cortical surface area, and cortical volume is described in the S1 Methods section online. The cortical regions of interest (ROIs) used to restrict the statistical analysis to brain structures that are part of the somatosensory-motor network are shown in S1 Fig online.

### Statistical analyses

#### Demographic and global brain measures

For the between groups comparison of demographic and global brain measures t-tests for independent samples were applied using IBM SPSS statistics version 22 (SPSS, an IBM company, Armonk, New York). Error probability was set at p < 0.05 (two-tailed) uncorrected for multiple comparisons.

#### Voxel-based morphometry and diffusion tensor imaging data

For the VBM and DTI data, group comparisons as well as correlations have been performed. Voxel-wise general linear models were applied using permutation-based non-parametric testing that also corrects for multiple comparisons across space (FSL’s randomise tool). The threshold free cluster enhancement technique was used in addition [62]. Error probability was set at p < 0.05 corrected for multiple comparisons using 5000 permutations in the group comparisons as well as in the correlation analyses.

We also correlated age of handball training commencement and years of handball training experience with the VBM and DTI parameters within the group of handball players. For these two correlations, data have to be demeaned using the—D option in randomise.

As described above, we restricted the statistical analyses of the VBM data to the regions of interest, i.e., to areas that are part of the somatosensory-motor network, and those of the DTI data to the CST and corpus callosum.

#### Subcortical volumetric segmentations

For the between-groups comparisons of the volumetric subcortical segmentations (basal ganglia, thalamus, corpus callosum, and cerebellum) analysis of covariance models were applied using IBM SPSS statistics version 22. In all these comparisons, total GM volume was used as a covariate of no interest in order to correct for global brain size differences. Error probability was set at p < 0.05 (two-tailed) uncorrected for multiple comparisons.

#### Associations among neuroplastic alterations

Associations between the VBM and DTI findings, subcortical, callosal, and cerebellar volumes were investigated using Pearson’s correlation implemented in IBM SPSS statistics version 22. Error probabilities was set at p < 0.05 uncorrected for multiple comparisons.

#### Surface-based morphometry data

For the SBM data, a vertex-wise general linear model based on parametric statistics was applied within FreeSurfer (MRI_GLMFIT tool) without using the threshold free cluster enhancement technique. Although the SBM data reported in the present study, the results of which are reported in the S1 Results section online, were not corrected for multiple comparisons, we indicate the clusters that would survive a Monte Carlo simulation based on the cluster extent using FreeSurfer’s MRI_GLMFIT-SIM tool applying 5000 permutations. Error probability was set at p < 0.05 in both the uncorrected and corrected analysis. In the uncorrected analysis, we additionally applied a cluster extent threshold that only considers clusters larger than 25 mm^2^ in size. Correlations between handball training-related measures and SBM-derived parameters were not conducted.

## Results

Demographic, behavioural, and global brain measures of the subjects under investigation are summarised in Table 2. There were no significant differences between handball players and control women with respect to age, intracranial volume, left or right total cortical GM volume, left or right total cortical WM volume, left or right total mid-cortical surface area, and left or right average cortical thickness. The mean, standard deviation, minimum, and maximum values of these measures as well as age of handball training commencement and years of handball training can be found in Table 2.

**Table 2:** Demographics, behavioural, and global brain measures of the handball players and the control women.

	Handball players (n = 11)				Control women (n = 12)				Significance
Measure	Mean	SD	Min.	Max.	Mean	SD	Min.	Max.	p-value
Age (years)	23.6	2.91	19.0	29.0	25.5	2.94	20.0	30.0	0.12
Age at handball training commencement	11.1	2.51	7.0	15.0	-	-	-	-	-
Number of handball training years	12.5	2.70	7.0	16.0	-	-	-	-	-
Intracranial volume (cm^3^)	1,388	128	1,041	1,529	1,376	126	1,144	1,580	0.55
Total left cortical grey matter volume (cm^3^)	243.2	11.8	221.9	260.9	234.5	12.6	213.6	255.8	0.11
Total right cortical grey matter volume (cm^3^)	242.7	12.9	222.7	258.6	233.4	12.1	209.0	257.8	0.09
Total left cortical white matter volume (cm^3^)	217.7	17.5	192.4	249.9	209.1	16.7	175.6	231.3	0.24
Total right cortical white matter volume (cm^3^)	220.2	16.6	193.1	251.1	210.2	17.2	179.5	232.6	0.17
Total left cortical surface area (cm^2^)	913.2	42.6	807.8	974.5	883.0	40.8	802.8	942.2	0.10
Total right cortical surface area (cm^2^)	921.4	41.5	820.4	970.0	887.1	47.8	785.3	949.1	0.08
Average left cortical thickness (mm)	2.613	0.085	2.509	2.792	2.609	0.055	2.531	2.725	0.91
Average right cortical thickness (mm)	2.583	0.089	2.471	2.722	2.582	0.044	2.500	2.655	0.98

n, number of subjects; Max., maximum; Min., minimum; p-value, error probability; SD, standard deviation.

In what follows, we first describe the results of the VBM and those of the DTI analysis. We then report the findings of the subcortical (basal ganglia and thalamus) volume analysis, those of the analysis of regional corpus callosum volumes based on T1-weighted MRI scans, and the findings of the cerebellar GM and WM volume analysis. Next, associations between the above-mentioned findings are described. Last, we briefly report what have been found in the SBM analysis, the results of which are only presented in the Supplementary Information online.

In addition to the results reported here, which all are statistically significant at a corrected alpha error probability, we also present findings derived from an explorative approach using a more liberal statistical threshold (0.05 < p < 0.10, corrected or uncorrected for multiple comparisons). All theses explorative findings, however, are not part of the main manuscript and can be found in the S1 Results section online.

### Voxel-based morphometry

**Fig 1:**
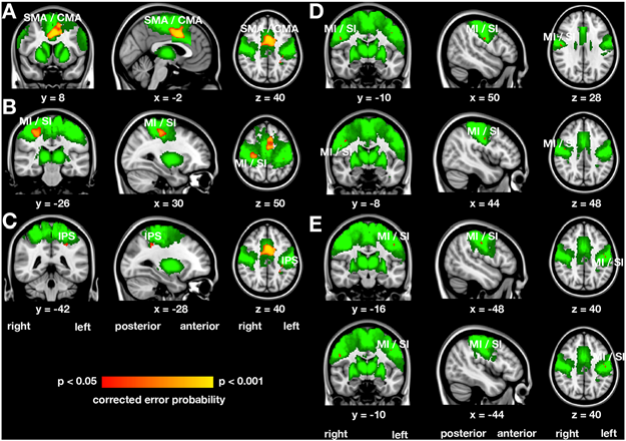
Regions with altered probabilistic grey volume in the somatosensory-motor cortex of handball players derived from the voxel-based morphometric group analysis (A-C) and the correlation analysis (D and E). The statistical parametric maps (shown in red yellow) were height-thresholded at p < 0.05 corrected for multiple comparisons using permutation-based non-parametric testing across space (FSL’s randomise tool, 5000 permutations). Increased grey matter volume was evident (A) in the bilateral supplementary motor area (SMA) and cingulate motor area (CMA) and (B) in the right primary motor and somatosensory cortex (MI and SI) and (C) in the left intraparietal sulcus (IPS). Age of handball training commencement was negatively correlated with grey matter volume in four small clusters, two clusters located in the right MI/SI region (D) and two clusters located in the left MI/SI region (E). The regions of interest subjected to the statistical analysis are shown in graded green. x, y, z represent coordinates of the Montreal neurological institute (MNI) stereotactic space.

The analysis of probabilistic cortical GM volume derived from the VBM data within the a priori defined regions revealed three clusters with increased cortical volume in handball players compared with control women (Fig 1A–1C and Table 3). The largest of these three clusters is located in the SMA/cingulate motor area (Fig 1A). The voxel with the local maxima is located in the left hemisphere, but the cluster extents also into the right hemisphere. The second cluster is located in the right MI/SI (Fig 1B) and the third cluster is located in the left intraparietal sulcus (Fig 1C).

**Table 3:** Regions with altered probabilistic grey volume in the somatosensory-motor cortex of handball players derived from the voxel-based morphometric group and correlation analysis.

	Letter in	Number of	Error	MNI coordinates		
Name (abbreviation)	Fig 1	voxels	probability	x	y	z
***Increased grey matter volume in handball players compared with control women***						
Left supplementary / cingulate motor area (SMA / CMA)	A	1446	0.005	-2	8	40
Right primary motor / somatosensory cortex (MI / SI)	B	316	0.019	30	-26	50
Left intraparietal sulcus (IPS)	C	85	0.040	-28	-42	40
***Negative correlation between grey matter volume and age of handball training commencement***						
Right primary motor / somatosensory cortex (MI / SI)	D	9	0.037	50	-10	28
Right primary motor / somatosensory cortex (MI / SI)	D	5	0.047	44	-8	38
Left primary motor / somatosensory cortex (MI / SI)	E	3	0.044	-48	-16	40
Left primary motor / somatosensory cortex (MI / SI)	E	1	0.047	-44	-10	40

MNI, Montreal neurological institute.

After correction for multiple comparisons, there were no clusters with decreased probabilistic GM volume in handball players compared with control women. No significant differences between the groups were found within the primary and secondary visual cortex (BA17 and BA18), which served as a negative control region.

When applying a whole brain group comparison no additional, statistically significant clusters were found. We then extracted the mean GM probabilistic volume within the three clusters revealed by the group comparison (Fig 1A–1C, Table 3) and correlated it with age of handball training commencement and years of handball training experience. Mean GM probabilistic volume was significantly inversely correlated with age of handball training commencement (r = -0.64, p = 0.035, n = 11), but not related to years of handball training experience (r = 0.25, p = 0.46).

However, within the group of handball players, we correlated age of handball training commencement and years of handball training experience with local GM volume also in voxel-wise analyses within the regions of interest (see section voxel-based morphometry in the materials and method section above). Four clusters with negative correlations between probabilistic GM volume and age of handball training commencement have been found. The two larger clusters were located within the right SI/MI region (Fig 1D and Table 3), whereas the two smaller clusters were located within the left SI/MI region (Fig 1E and Table 3). There were no significant correlations between years of handball training experience and local GM volume. Although these four clusters are actually too small to be commonly reported (only between 9 and 1 voxels in size), we nevertheless report these clusters because no arbitrary cluster extent threshold has to be applied when using the threshold free cluster enhancement technique [62].

### Diffusion tensor imaging

**Fig 2:**
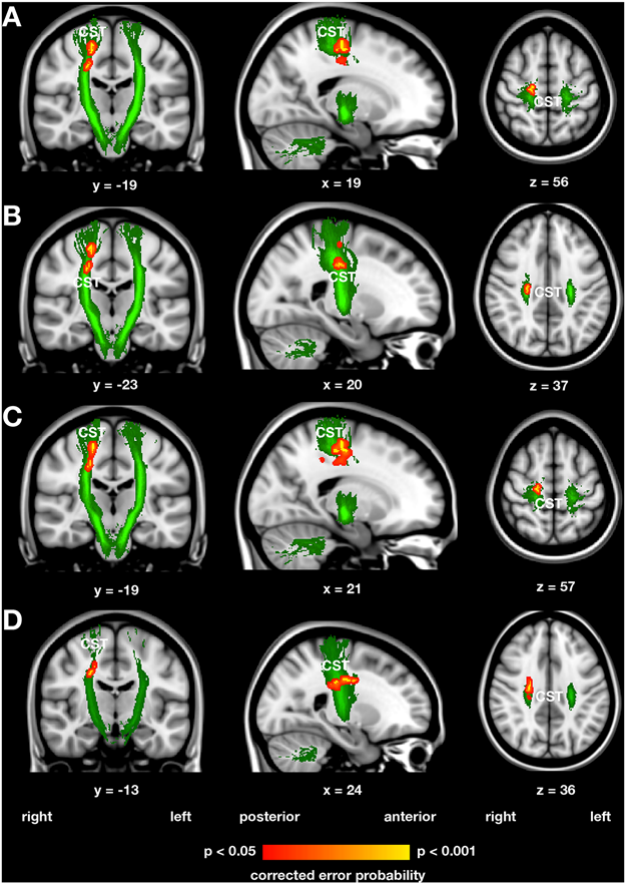
Increased fractional anisotropy and axial diffusivity in the right corticospinal tract of handball players derived from the tract-based spatial statistics group analysis (A and B) and correlation analysis (C and D). The statistical parametric maps (shown in red yellow) were height-thresholded at p < 0.05 corrected for multiple comparisons using permutation-based non-parametric testing across space (FSL’s randomise tool, 5000 permutations). Increased fractional anisotropy (FA) was evident in the corticospinal tract (CST) immediately inferior to the right premotor and primary motor region (Fig 2A) and approximately on the level of the corpus callosum (Fig 2B). The analysis of axial diffusivity revealed that increased FA in the CST of handball players (Figs 2A and 2B) is mainly driven by increased axial diffusivity (Fig 2C). Years of handball training experience were inversely associated with radial diffusivity in a cluster located in the right CST on the height of the corpus callosum (Fig 2D). The corpus callosum is not shown because the finding in that structure was only significant at a trend level towards statistical significance. The regions of interest, here the left and right CST, subjected to the statistical analysis are shown in graded green. x, y, z represent coordinates of the Montreal neurological institute (MNI) stereotactic space.

The analysis of the diffusivity indices derived from the DTI data revealed three clusters with increased FA and one cluster with increased axial diffusivity within the right CST in handball players compared with control women (Fig 2 and Table 4). The largest of the three FA clusters is located immediately inferior to the right premotor and MI region (Fig 2A). The second largest cluster is located inferiorly to the largest one, i.e. approximately on the level of the corpus callosum (Fig 2B).

**Table 4:** Regions with increased fractional anisotropy and axial diffusivity in the corticospinal tract in handball players derived from the diffusion tensor imaging analysis.

	Letter in	Number of	Error	MNI coordinates		
Name (abbreviation)	Fig 1	voxels	probability	x	y	z
***Increased fractional anisotropy in handball player compared with control women***						
Right corticospinal tract (CST in motor / premotor regions)	A	129	0.012	19	-19	56
Right corticospinal tract (CST near corpus callosum)	B	80	0.030	23	-20	37
Right corticospinal tract (CST near corpus callosum)	-	1	0.049	24	-23	32
***Increased axial diffusivity in handball players compared with control women***						
Right corticospinal tract (CST in motor / premotor regions)	C	325	0.030	19	-21	57
***Negative correlation between radial diffusivity and years of handball training experience***						
Right corticospinal tract (CST near corpus callosum)	D	163	0.020	24	-13	36
Right corticospinal tract (CST near corpus callosum)	-	6	0.049	20	-15	43
Right corticospinal tract (CST near corpus callosum)	-	1	0.049	20	-13	41

MNI, Montreal neurological institute.

Although the third FA cluster is actually too small to be commonly reported (only 1 voxel in size), we nevertheless report it because no (arbitrary) cluster extent threshold has to be applied when using the threshold-free cluster enhancement technique [62]. The analysis of axial diffusivity revealed that increased FA in the CST of handball players (Figs 2A and 2B) is mainly driven by increased axial diffusivity (Fig 2C).

There was no significant cluster that showed decreased mean diffusivity in handball players. After correction for multiple comparisons, there were neither clusters with increased FA or axial diffusivity nor clusters with decreased mean or radial diffusivity in control women compared with handball players.

With respect to the corpus callosum and after correction for multiple comparisons, there was no significant cluster that showed differences between groups in FA, mean, axial, or radial diffusivity. No significant differences between the groups were found in any of the diffusivity parameters within the cingulum, which served as a negative control tract.

**Fig 3:**
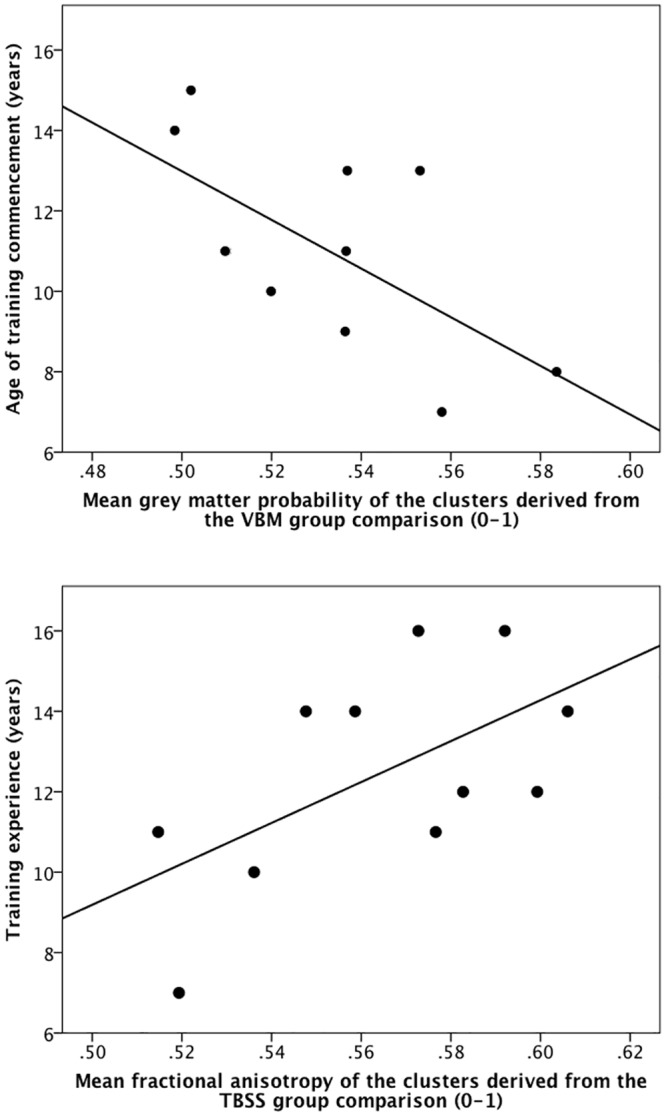
Associations between handball training-related measures and grey and white matter characteristics. Illustrated are the correlation between age of training commencement and mean grey matter density within the clusters revealed in the VBM group comparison (r = -0.64, R^2^ = 0.41, p = 0.018, one-tailed, n = 11, upper panel) as well as the correlation between years of training experience and mean fractional anisotropy within the clusters revealed in the TBSS group comparison (r = 0.59, R^2^ = 0.35, p = 0.027, one-tailed, n = 11, lower panel).

We then extracted the mean FA within the three clusters revealed by the FA group comparison (Figs 2A and 2B and Table 4) and mean axial diffusivity within the cluster revealed by the axial diffusivity group comparison (Fig 2C and Table 4) and correlated these diffusivity values with age of handball training commencement and years of handball training experience. Mean FA was positively correlated with years of handball training experience (r = 0.59, p = 0.027 one-tailed, n = 11, see also Fig 3), whereas mean FA was not related to age of handball training commencement (r = -0.35, p = 0.29). Mean axial diffusivity was neither related to years of handball training experience (r = 0.07, p = 0.84) nor to age of handball training commencement (r = -0.43, p = 0.19).

However, within the group of handball players, we correlated age of handball training commencement and years of handball training experience with local FA, mean, axial and radial diffusivity also in voxel-wise analyses within the regions of interest, i.e. within the left and right CST. Years of handball training experience were inversely associated with radial diffusivity in a cluster located in the right CST on the height of the corpus callosum (Fig 2D, and Table 4). Age of handball training commencement was not associated with any of the four diffusion indices.

Fig 3 illustrates the association between age of training commencement and mean GM density within the clusters revealed in the VBM group comparison as well as the association between years of training experience and mean FA within the clusters revealed in the TBSS group comparison.

### Subcortical volumes

Subcortical brain volumes of the handball players and control women are summarised S2 Table. A multivariate analysis of covariance (MANCOVA) correcting for total GM volume revealed significantly increased volumes of all basal ganglia structures (putamen, caudate nucleus, pallidum) and the thalami together (multivariate) in professional handball players compared with control women (F_(8,13)_ = 5.88; p = 0.003; ƞ_p_^2^ = 0.78). Subsequent post hoc univariate analyses of covariance (ANCOVA) were statistically not significant (all error probabilities p > 0.063, uncorrected). There was no significant correlation between years of handball training or age of training commencement and the volumes of the eight subcortical structures.

### Regional corpus callosum volumes

A MANCOVA also corrected for total GM volume revealed no significant volume differences of all five corpus callosum regional volumes together (F_(5,16)_ = 1.01; p = 0.44; ƞ_p_^2^ = 0.24). Subsequent post hoc ANCOVAs corrected for total GM volume revealed no statistically significant results. There was no significant correlation between age of training commencement or years of handball training and the volume of the five callosal subregions and the sum of them.

### Global cerebellar grey and white matter volumes

With respect to the cerebellum, an ANCOVA corrected for total GM volume revealed no statistically significant difference in cerebellar GM volume. However, handball players showed significantly reduced cerebellar WM volumes compared with control women (left hemisphere: F_(1,20)_ = 7.65; p = 0.012; ƞ_p_^2^ = 0.28; right hemisphere: F_(1,20)_ = 5.14; p = 0.035; ƞ_p_^2^ = 0.20) (S2 Table).

There were no significant correlations between age of training commencement or years of handball training and the left or right cerebellar GM or WM volume.

### Associations among neuroplastic alterations

We also investigated whether associations exist among the neuromorphometric features of different regions of interest. For that purpose, we first extracted mean GM probability of the clusters revealed in the VBM analysis and mean FA and axial diffusivity of the clusters revealed in the DTI analysis and then correlated these measures with each other as well as with subcortical, callosal, and cerebellar volumes, the latter of which are also correlated with each other. Due to the fact that the two groups differ in most of these measures (as shown above), one cannot investigate the associations of these neuroplastic alterations across both groups because this would be an issue of “double dipping” and would result in inflated correlation coefficients [63]. To avoid this issue, correlations have been applied within each group separately. The correlation coefficients and error probabilities of these associations are summarized in Table 5.

**Table 5:** Associations among neuroplastic alterations.

		Mean value within clusters			Cerebellum		Corpus callosum	Left subcortical	Right subcortical
Measures	Tissue	Grey matter	Fractional anisotropy	Axial diffusivity	Grey matter	White matter	White matter	Grey matter	Grey matter
**Mean value within clusters**	**Grey matter**	**-**	-0.04	-0.13	-0.29	-0.44	-0.15	-0.06	0.03
		**-**	0.91	0.71	0.38	0.19	0.67	0.87	0.94
	**Fractional anisotropy**	-0.27	-	**0.62**	0.38	0.02	**0.76**	0.52	0.40
		0.40	-	**0.04**	0.25	0.96	**0.006**	0.11	0.22
	**Axial diffusivity**	-0.03	**0.83**	-	0.52	0.43	0.55	**0.67**	**0.61**
		0.92	**0.001**	-	0.10	0.19	0.078	**0.026**	**0.046**
**Cerebellum**	**Grey matter**	-0.27	0.16	-0.13	-	**0.80**	0.49	**0.64**	0.53
		0.40	0.63	0.69	-	**0.003**	0.12	**0.035**	0.090
	**White matter**	0.18	0.43	0.34	0.47	-	0.37	**0.65**	0.60
		0.85	0.16	0.28	0.12	-	0.27	**0.030**	0.054
**Corpus callosum**	**White matter**	-0.15	0.32	0.37	0.15	0.19	-	**0.74**	**0.69**
		0.65	0.31	0.24	0.64	0.55	-	**0.010**	**0.020**
**Left subcortical**	**Grey matter**	0.15	0.16	0.24	0.50	0.48	**0.67**	-	**0.97**
		0.65	0.63	0.45	0.10	0.11	**0.018**	-	**4.44E-07**
**Right subcortical**	**Grey matter**	0.04	0.13	0.20	0.50	0.42	**0.72**	**0.98**	-
		0.91	0.69	0.54	0.10	0.17	**0.008**	**3.93E-08**	-

The right-sided upper triangular matrix represents the correlations of the measures in the handball players and the left-sided lower triangular matrix represents the correlations of the measures in the control group. The first row per tissue represents the Pearson’s correlation coefficient and the second row the corresponding p-value. Statistically significant correlations are printed in bold.

Of the 28 correlations conducted within the group of control women only 4 correlations reached statistical significance, two of which were a prior expected and declared as not interesting, i.e. the correlation between FA and axial diffusivity as well as the correlation between left and right subcortical volumes. In contrast, 10 out of 28 correlations conducted within the group of professional handball players reached statistical significance. Mean FA of the clusters found in CST with the DTI analysis is positively correlated with corpus callosum white matter volume derived from the T1-weighted image. The left and in part the right subcortical volumes derived from the T1-weighted image positively correlated with axial diffusivity in the CST, cerebellar GM and WM volume and corpus callosum volume, the former measure derived from the DTI analysis and the latter measures derived from the T1-weighted image. None of the negative associations reported in Table 5 reached statistical significance.

### Surface-based morphometry

As already mentioned, the surface-based analysis served as a replication and validation approach and its results are summarised in S1 Table and presented in S2–S4 Figs. In summary, the regions that have been shown to be altered between groups in the VBM analysis of probabilistic GM volume, i.e., the MI/SI hand representation and the SMA, revealed also differences in cortical GM volume derived from the SBM approach (S2 Fig). In addition to these replicated findings, the right premotor cortex showed increased cortical volume (S2 Fig) that is driven by increased cortical surface area (S3 Fig), whereas cortical thickness was reduced in the left premotor cortex (S4 Fig). Please note that these surface-based findings should be interpreted with caution due to the fact that most of these clusters did not survive correction for multiple comparisons. More details with respect to the SBM replication and validation approach can be found in the S1 Methods and S1 Results section online.

## Discussion

In the present study, we investigated specific anatomical features of the brain of professional female handball players compared with control women without a history of any ball-related sport experience and/or activity. By using two different MRI modalities (T1- and diffusion-weighted) combined with different computational, neuromorphometric analysis tools (VBM, SBM, TBSS, subcortical volumetric segmentation) we were able to explore in greater detail the neural correlates of training-induced neuroplastic adaptations in handball players. In the case of GM morphology, we focused on the hand representations in MI and SI, but other GM regions of the somatosensory-motor network such as the SMA, basal ganglia, thalamus, and cerebellum were investigated in addition. In the case of WM morphology, we focused on the diffusion properties of the CST and corpus callosum in our analyses based on DTI data, while callosal and cerebellar WM volumes based on T1-weighted images were investigated as well.

As predicted, we found differences in GM and WM morphology between handball players and control subjects in brain regions associated with the motor and somatosensory control of both hands (and arms). Main results are increased GM volume in handball players in the right MI/SI hand representation, in the SMA / cingulate motor area (this cluster extends across both hemispheres) and in the left intraparietal sulcus as well as increased FA and axial diffusivity in the right CST, which is involved in the motor control of the left hand and arm. Age of handball training commencement is inversely related to GM volume in the right and left MI/SI hand representation, whereas years of handball training experience positively correlates with mean FA within the right CST. Alterations in GM morphology are associated with alterations in white matter morphology.

Most of the studies listed in our review of the literature (see Table 1) investigated only one brain structure of the somatosensorimotor network and also applied only one imaging approach, e.g., Park and colleagues investigated either the cerebellar vermis or the putamen of basketball players using manual volumetry based on T1-weighted MRI scans [25,26]. Most of these studies therefore provide only limited information with respect to the plastic changes associated with the different types of sports.

However, the core value of the present study is its more holistic and complementary approach that is characterized by (I) the investigation of almost all brain structures associated with the somatosensorimotor network, (II) the application of different MRI modalities to investigate GM and WM anatomy simultaneously, (III) the combination of different computational neuromorphometric tools and analyses, (IV) the direct association of the GM and WM results as well as (V) the fact that the study also provides a review of the literature about structural brain correlates associated with other forms of sport. The main findings of the present study are discussed in greater detail below.

### Grey matter differences in the primary motor and primary somatosensory cortex, supplementary motor area, and premotor cortex

When comparing our results with those reported by other structural imaging studies that also investigated sportsmen (see Table 1), convergent findings emerged. Jacini and colleagues investigated judo wrestlers by applying VBM and also found increased GM volume in the left MI, left SI, and bilateral SMA in judo wrestlers compared with controls [23]. World-class gymnasts were investigated using VBM and DTI and this study revealed increased GM volume in the right MI and right SI of the gymnasts, but gymnasts showed decreased FA in the white matter underlying the MI (most probably representing the fibres of the CST), SI, SMA, and premotor cortex mainly in the left hemisphere [21]. In both studies, the clusters in MI and SI are approximately located in the MI and SI hand representation, respectively. Using the same experimental subjects as investigated by Huang and colleagues (2013), Wang and colleagues applied graph-theoretical (network) analyses based on structural connections derived from DTI data and showed that the local network efficiency is enhanced in MI (most probably representing the fibres of the CST) and SI in gymnasts compared with control subjects [28].

Although we did not find statistically significant GM differences in the premotor cortex when correcting for multiple comparisons in the VBM analysis, we found evidence for neuroplastic alterations of the premotor cortex in handball players using our surface-based morphometric approach. Cortical volume and cortical surface area were increased in the right premotor cortex (S2 and S3 Figs), whereas cortical thickness was decreased mainly in the left premotor cortex of the handball players compared with control women (S4 Fig). However, only the left premotor cluster with reduced cortical thickness would survive correction for multiple comparisons. Therefore, these premotor findings have to be interpreted with caution.

Handball playing is very similar to basketball playing, but unfortunately the three studies listed in Table 1 that investigated basketball players did only investigate the cerebellum and basal ganglia [24–26] and did not provide any information with respect to MI, SI, SMA, and the premotor cortex. Although it is obvious that bimanual hand movements are central for playing handball and structural brain alterations in MI and SI hand representations are hence to be expected, it remains unclear why such differences are also predominant in judo wrestlers [23] and world-class gymnasts [21,28]. This is because alterations in GM of MI and SI other than the hand representations might be also plausible for wrestlers and gymnasts. When considering the demands on judo wrestlers with respect to the fingering, i.e. reaching the opponent’s judo outfit as well as considering the disciplines of the gymnasts that included, among others, pommel horse, still rings, parallel bars, horizontal bar, and uneven bars, all gymnastic disciplines for which the hands are more central than it would be the case for vault, floor exercise, and balance beam, disciplines that are underrepresented among the gymnasts investigated in these studies [21,28].

### Morphological differences in the corticospinal tract

Morphological alterations in the handballer’s brain are not restricted to the GM morphology. The WM morphology is altered as well. Two clusters with increased FA and axial diffusivity within the handballer’s right CST were found. When comparing our results with those reported by other structural imaging studies of sportsmen based on DTI data (see Table 1), convergent but also divergent findings have been reported. Increased FA has also been observed in the CST of world-class gymnasts [28], but reduced FA in the CST and internal capsule has been found in golfers [17], whereas no FA differences in the CST and internal capsule have been reported for ballet dancers [16]. But in these two studies of our group, we found clusters with decreased WM volume in the CST and in the internal and external capsule of both professional ballet dancers [16] and golfers [17]. However, the relationship between WM volume and FA derived from MRI scans remains to be further investigated, so far only weak associations between these two measures have been reported [64,65]. The only longitudinal study reported in our literature review (Table 1) investigated mountaineers who underwent a 30-days expedition to the Tanggula mountains (6,206 metres) at Qinghai-Tibet-Plateau using DTI [31]. Although this study reported decreased FA in several fibre tracts including the CST, its findings should be interpreted in the context of high-altitude exposure rather than in the context of structural neuroplasticity evoked by the climbing activity itself.

### Volumetric differences of the basal ganglia and thalamus

The findings of the analysis of the subcortical volumes should be interpreted with caution because they only reach statistical significance when analysed in a multivariate way, i.e. both thalami and all bilateral basal ganglia substructures together. Although all structures tended to be larger in handball players (S2 Table), only the right caudate nucleus showed a larger volume in handball players compared with control women and age of handball training commencement was inversely related to the volume of the left caudate nucleus. Although the two results are statistically significant only on trend level these effects might suggest that the caudate nucleus is more important than other basal ganglia structure or even more important than the thalamus for playing handball on a high level. Park and colleagues investigated the striatum of professional basketball players using manual volumetry and this study revealed increased volumes of both caudate nuclei and both putamina in basketball players compared with control men [26]. In contrast, using VBM reduced GM volumes were found in the left putamen of professional female ballet dancers [16] as well as in the left caudate nucleus of high diving players [29]. However, compared with control subjects the bilateral thalamus in high diving players showed increased GM volume [29] and in the same sample of sportsmen the bilateral thalamus and left pallidum were regionally inflated in high diving players [32]. Increased thalamic GM volume has been found in slackliners / dancers [22] and enhanced thalamic local network efficiency has also been reported for world-class gymnasts [28]. A poster presented at the 43^rd^ Annual Meeting of the Society for Neuroscience, San Diego, CA, USA, November 09–13, 2013) showed that left and right ventral striatal volumes are enlarged in two internationally known top track and field athletes, a male javelin thrower and a male long jumper [66]. These authors concluded that the neuroplastic changes in the ventral striatum might be related to the mental effort of long-term deliberate practice [67] rather than related to the motor and somatosensory aspects and demands of the sport itself, which are more strongly associated with the dorsal striatum compared with the ventral striatum.

### Volumetric differences of the corpus callosum and cerebellum

The sum of all five corpus callosum regional volumes tended to be larger in handball players than in control women (S2 Table). Unfortunately, the studies summarised in our literature review (see Table 1) did not investigate the corpus callosum at all or did report a negative callosal finding. However, reduced WM volume of the corpus callosum has been reported for professional ballet dancers [16] as well as for golf players [17]. When looking at particular regional callosal volumes, the posterior and mid-posterior callosal portion tended to be larger in handball players compared with control women, albeit only on a trend level toward statistical significance. Nevertheless, transcallosal fibres connecting homolog primary motor and primary somatosensory cortices, regions which are altered in handball players (see above), pass through the corpus callosum in its posterior and mid-posterior portions [68]. Exactly these posterior and mid-posterior callosal regions are larger in the handball players investigated in the present study, albeit only on a trend level towards statistical significance (p = 0.097 and p = 0.060, respectively).

With respect to the cerebellum, handball players showed larger cerebellar GM volumes, but only on a trend level, whereas their cerebellar WM volumes were statistically significantly reduced compared with control women (S2 Table). Using manual volumetry, the cerebellum as a whole did not differ significantly between basketball players and control subjects [24], but showed increased GM and WM volumes of the vermal cerebellar cortex when the basketball players’ cerebellum was divided into substructures and investigated in more detail [25]. Enhanced cerebellar GM and/or WM volumes have also been found in speed skaters [27], badminton players [20], slackliners / dancers [22], and in professional climbers as well [19].

### Associations between neuroplastic measures

The analysis of the associations between the various measures investigated in the present study aimed at highlighting whether the alterations observed were independent from each other or whether alterations in one brain region are related to alterations in other brain regions. The statistically significant associations found between the different morphological measures were all positive (see Table 5). These correlations suggest that the structural measures of the different neuroanatomical features and regions are related in handball players, but are unrelated in control women. These positive associations suggest that some of the alterations we observed in the handballer’s brain are non-independent. The absence of negative associations among the region of interest investigated in the present study suggests that alterations in one brain region are not implemented on the costs of contrary alterations in other brain regions. However, it might be the case that such redistributions of tissue exist, but involve brain regions not part of the somatosensory-motor network, i.e. regions that are part of other functional networks not investigated in the present study.

### Use-dependent versus deprivation-related neuroplastic alterations

Use-dependent neuroplastic changes more often lead to increases than to decreases in tissue and can therefore be denoted progressively in its nature and might be regarded as a “mirror finding” of what have been reported in another study that investigated the effects of short-term upper limb immobilization on brain plasticity and these changes can be denoted regressively (deprivation-related) neuroplastic alterations [69]. In that study, 10 right-handed subjects with injury of the right upper extremity that required at least 14 days of limb immobilization have been investigated. Subjects underwent 2 MRI examinations, the first within 48 hours post-injury and the second after an average time interval of 16 days of limb immobilization. After immobilization, a decrease in cortical thickness in the left primary motor and somatosensory cortex and a decrease in FA in the left CST has been revealed. These left-hemispheric structures control the right, injured extremity. In addition, the motor skill of the left (non-injured) hand improved and is related to increased cortical thickness in the right motor cortex and increased FA in the right CST [69]. This study shows that neuroplastic changes can occur within a time interval of only 16 days, that alterations are regressive (decrease in volume, thickness, or FA) in the case of reduced activity (immobilization), and that alterations are progressive (increase in volume, thickness, or FA) in the case of enhanced activity. Further evidence that deprivation-related (regressive) and use-dependent (progressive) plasticity goes hand in hand has recently been reported for limb amputees too [70]. Long-term intensive handball training is clearly an example of enhanced activity and our findings of increased GM volume in the left and right MI/SI hand representation and increased FA in the right CST are thus in line with the findings of the two longitudinal studies reported above [69,70].

### Voxel-based versus surface-based morphometry

When comparing the results derived from the VBM analysis with those derived from the SBM analysis mainly convergent findings emerged. For example, the cluster with increased probabilistic GM volume found in the SMA/CMA region in the VBM analysis (MNI coordinates: x = -2, y = 8, z = 42, see Fig 1A and Table 2) has also been revealed by the SBM analysis of cortical volume (x = -8, y = 13, z = 48, see SMA/CMA label in S2 Fig and letter F in S1 Table). Subsequent analyses of cortical surface area and thickness revealed that the volume increase in this SMA/CMA cluster is solely driven by increases in cortical surface area (x = -9, y = 15, z = 54, see S3 Fig and letter F in S2 Table) and not by increases in cortical thickness (x = -9, y = 12, z = 58, see S4 Fig and letter F in S2 Table). Moreover, cortical thickness in this region was reduced in handball players compared with control women. Similar patterns of morphological changes between handball players and control women were found for other clusters as well.

There are studies that also reported convergent findings between the VBM and SBM methodology [71–73], but other studies showed divergent findings between the two methodologies [74,75]. There is also a report that compared VBM with a voxel-based cortical thickness method revealing consistent results between both methodologies [76]. But it is important to note that this voxel-based cortical thickness analysis is not really a surface-based procedure embedded in a geodesic coordinate system that would take into account the geometry of the cortex. The pre-processing steps of this voxel-based cortical thickness analysis is identical with the pre-processing steps applied in a classical VBM; hence, this might explain the almost identical results obtained from these two morphometric analyses [76].

The potential major source of variability between the results derived from the VBM approach compared with those derived from the SBM approach might originate from differences in the methodologies itself. E.g. VBM is implemented in a Cartesian coordinate system based on voxels and measures probabilistic GM volume derived from one 3D brain model, whereas SBM is implemented in a geodesic coordinate system based on vertices and measures cortical thickness and surface area derived from several 2D surface models that are in spatial correspondence. A potential further source of variability might arise from the fact that if cortical thickness and cortical surface area differences in local tissue points in different directions [77,78], effects can be detected with the SBM methodology, but not with the VBM technique. This is because the two measures are mixed in the concept of probabilistic GM volume used by VBM and therefore the effect of one measure tends to cancel out the effect of the other measure. A potential minor source of variability between the results derived from the VBM compared with those derived from the SBM approach might originate from differences in spatial smoothing procedures. VBM commonly use 3D voxel-based blurring smoothing kernels (mostly Gaussian kernels) that do not follow the topology and geometry of the cortex, whereas surface-based morphometric models are commonly smoothed with 2D vertex-based surface diffusion smoothing kernels that follow the topology and geometry of the cortex. The former smoothing procedure mixes up information from different functional brain regions that are far away on the cortex, but in direct neighbourhood in the voxel-based space of the brain model, whereas 2D vertex-based surface diffusion smoothing kernels operate on the cortical surface of the digital brain models and therefore do not mix up information from different functional brain regions [79,80]. Although some advantages of SBM compared with VBM are reported here, there is no consent about when one should use SBM and in which cases VBM is the preferred methodology.

### Underlying cellular mechanisms of neuroplastic alterations

Although structural neuroplastic alterations can be measured with MRI at the macroscopic scale, the underlying cellular events of these changes at the microscopic scale are not clear yet. Several mechanisms underlying these microscopic changes have been proposed [1,2]. The macroscopic changes may be attributed to an increase in cell size, genesis of new synapses, genesis of glial or even neural cells, or changes in spine density, blood flow, interstitial fluid, or even angiogenesis. However, given that current knowledge regarding cellular and physiological mechanisms is indeed marginal, it is simply impossible to provide convincing micro-scale explanations on the basis of our MR signal. This knowledge is necessary in order to make inferences about the possible physiological consequences of these cellular alterations.

### Limitations

Several limitations of the present study are worth mentioning. First, the sample sizes in the present study were rather small (11 handball players versus 12 control women) and might therefore diminish the reliability to detect between group differences. Although this might be a major limitation of the present study (and most of all neuroimaging studies) it is clear that strong effects identified with small samples will also be identified in larger samples. When using corrected statistical tests as have been applied in our present study the alpha error is small, but the beta error becomes large. Thus, meaningful moderate or small effects will only be detected with larger samples [81]. However, future investigations should recruit a larger number of experts and/or enhance the number of the control subjects both help increasing the statistical power, which should be estimated a priori with the help of a power analysis. Second, although we expected a superior left hand ability and performance with respect to catching and throwing of a ball in professional handball players compared with control women, we did not directly measure the left and right hand performance of the participants. Future studies should perform symmetric and asymmetric bimanual reaching tasks such as used in previously published studies [82], assessments used to test other professionals [83] or any in-house tailored handball playing test including catching and throwing abilities. Third, it remains unknown whether the structural alterations reported are sport-induced general adaptations or specific related to the bimanual nature of handball playing. Future studies should include an active control group of e.g. soccer players in order to distinguish between general sport-related from handball-related structural brain adaptations. Forth, although the sample sizes were rather small and hence limiting statistical power, the effects reported showed medium to large effect sizes. Fifth, whether the structural alterations found in handball players are the result of training-induced neuroplastic adaptations (nurture) or origin from a genetic predisposition (nature) for a “ball playing affinity” should be investigated in future longitudinal studies. Sixth, it remains unknown whether the results found for professional female handball players would also be representative for neuroplastic alterations of professional male handball players. Last, the literature review of neuromorphometric differences in sportsmen and sportswomen presented in the present study provide strong evidence for morphometric alterations in motor, somatosensory, SMA, and premotor cortex across a variety of different forms of sport. However, sport-general as well as specific contribution of one particular form of sport onto morphological alterations of dedicated cortical and subcortical brain structures remains to be investigated in future structural and functional imaging studies.

## Conclusions

As predicted, we found differences in GM and WM morphology between handball players and control women in brain regions associated with the motor and somatosensory control of the hands. Main results are increased GM volume in handball players in the right and left MI/SI hand representation, more extended and with a higher effect size in the right than left hemisphere, in the SMA/cingulate motor area (the cluster extends across both hemispheres) as well as increased FA and axial diffusivity in the right CST. Brain structures seem to be altered in handball players as a consequence of the intensive handball playing training rather than a consequence of a genetic predisposition for a particular neural trait because handball playing-related measures such as age of handball training commencement correlated negatively with GM volume in the right and left MI/SI and years of handball training experience correlated positively with FA in the right CST. However, longitudinal studies are needed in order to unequivocally track down whether the observed structural alterations are driven by nurture (trainings-induced neuroplasticity) or by nature (genetic predisposition) or potentially driven by both. Investigations of neuroplasticity specifically in sportsmen help to understand the neural mechanisms of expertise in general and might contribute to improvements in the field of neurorehabilitation as has been recently shown by other studies [84].

## Supporting Information

S1 Fig Regions of interest used in the surface-based morphometric analysis.
(DOCX)

S2 Fig Replication and extension of the voxel-based morphometric finding reported in the main manuscript. The surface-based morphometric approach also revealed increased cortical volume in professional handball players compared with control participants in identical, similar and other regions as found in the voxel-based morphometric analysis. The statistical parametric maps were height-thresholded at p < 0.05 (uncorrected for multiple comparisons) as well as cluster extent-thresholded by considering only clusters larger than 25 mm^2^. The left column shows the left hemisphere and the right column shows the right hemisphere. The first row represents lateral views of mean inflated surface models derived from the subjects under investigation and rotated by 30° in order to have a better view into the central sulcus. The second row represents the medial views. More detailed information of the clusters presented can be found in S1 Table. Abbreviations: CMA, cingulate motor area; MI, primary motor cortex; PMC, premotor cortex; SI, primary somatosensory cortex; SMA, supplementary motor area.
(DOCX)

S3 Fig Increased cortical surface area drives increased cortical volume in handball players. Statistical maps were thresholded with p < 0.05 (uncorrected for multiple comparison). The left column shows the left hemisphere and the right column shows the right hemisphere. The first row represents lateral views of mean inflated surface models derived from the subjects under investigation and rotated by 30° in order to have a better view into the central sulcus. The second row represents the medial views. More detailed information of the clusters presented can be found in S1 Table. Abbreviations: CMA, cingulate motor area; MI, primary motor cortex; PMC, premotor cortex; SI, primary somatosensory cortex; SMA, supplementary motor area.
(DOCX)

S4 Fig None, only marginal or inverse differences in cortical thickness in brain regions that showed increased cortical volume in professional handball players. The statistical parametric maps were height-thresholded at p < 0.05 (uncorrected for multiple comparisons) as well as cluster extent thresholded by considering only clusters larger than 25 mm^2^. A) Left hemisphere and B) right right hemisphere. The first row represents lateral views of mean inflated surface models derived from the subjects under investigation and rotated by 30° in order to have a better view into the central sulcus. The second row represents the medial views. The inset in A) represent the PMC cluster rotated down by 60° to reveal that this is one connected cluster. The inset in B) represent the SII cluster rotated up by 60° to reveal the whole extent of this cluster. More detailed information of the clusters presented can be found in S1 Table. Abbreviations: CMA, cingulate motor area; MI, primary motor cortex; PMC, premotor cortex; SI, primary somatosensory cortex; SII, secondary somatosensory cortex; SMA, supplementary motor area.
(DOCX)

S1 Methods Regions of interest used in the surface-based morphometric analysis. The clusters found in the voxel-based morphometric analysis are located within these regions of interest (ROIs). The exact boundaries of these ROIs can be found elsewhere (see supplementary reference #13 in S1 Methods). The left column shows the left hemisphere and the right column shows the right hemisphere. The first row represents lateral views of mean inflated surface models derived from the subjects under investigation and rotated by 30° in order to have a better view into the central sulcus. The second row represents the medial views. The functional brain areas located within these ROIs are reported in brackets. Abbreviations: CMA, cingulate motor area; MI, primary motor cortex; PMC, premotor cortex; SI, primary somatosensory cortex; SII, secondary somatosensory cortex; SMA, supplementary motor area.
(DOCX)

S1 Results Additional findings that were statistically not significant, but showed a trend toward significance (0.05 < p < 0.10).
(DOCX)

S1 Table Regions with increased or decreased cortical volume, surface area, and thickness in the somatosensory-motor network in handball players derived from the surface-based morphometric analysis. Clusters within the different measures are ordered by decreasing statistical significance. Bold printed clusters represent cluster within the predicted regions of interest (ROIs). The ROIs also contain parts of the brain for which we did not pose any hypothesis because these ROIs were chosen from a predefined atlas. However, the clusters found outside of the predicted regions but inside the ROIs are presented for completeness. (HABA > CON) indicates clusters that showed increased values in handball players compared with control women. (CON>HABA) indicates clusters that showed decreased values in handball players compared with control women. The statistical parametric maps reported here were height-thresholded at p < 0.05 (uncorrected for multiple comparisons) as well as cluster extent thresholded by considering only clusters larger than 25 mm^2^ in size. Clusters indicated by the paragraph sign (§) survived error correction for multiple comparisons by using Monte Carlo simulations on the cluster size using 5000 permutations. The ampersand sign (&) highlights clusters with cortical differences in handball players compared with controls as found by both the voxel-based as well as the surface-based morphometric analysis. (A) to (H) indicate corresponding clusters across cortical measures. Abbreviations: BA, Brodmann area; CMA; cingulate motor area; MI, primary motor area; MNI, Montreal neurological institute (space); PMC, premotor cortex; SI, primary somatosensory cortex; SII, secondary somatosensory cortex; SMA, supplementary motor area.
(DOCX)

S2 Table Volumes of subcortical structures including the corpus callosum and cerebellum derived from the volumetric segmentation analysis. *, corpus callosum volume was computed based on a mid-sagittal slice of 5 mm thickness.
(DOCX)
