# Supplementary material for: Structural Brain Correlates Associated with Professional Handball Playing
Source: PLoS One. 2015 Apr 27;10(4):e0124222. doi: 10.1371/journal.pone.0124222 (PMC4411074; doi:10.1371/journal.pone.0124222)
Supplement: S1 Fig — Regions of interest used in the surface-based morphometric analysis. (DOCX) [file pone.0124222.s001.docx]

**Structural Brain Correlates Associated with Professional Handball Playing**

Jürgen Hänggi^1*,#a^, Nicolas Langer^1-3^, Kai Lutz^1,4,5^, Karin Birrer^1,6^, Susan Mérillat^1,7^ and Lutz Jäncke^1,7-10^

^1^ Division Neuropsychology, Department of Psychology, University of Zurich, Zurich, Switzerland

^2^ Neural Systems Lab, The City College of New York, New York, NY, USA

^3^ Child Mind Institute, New York, NY, USA

^4^ Center for Neurology and Rehabilitation, cereneo AG, Vitznau, Switzerland

^5^ Department of Neurology, University Hospital Zurich, Zurich, Switzerland

^6^ Rehabilitation Center Affoltern am Albis, University Children’s Hospital Zurich, Affoltern am Albis, Switzerland

^7^ International Normal Aging and Plasticity Imaging Center (INAPIC), University of Zurich, Zurich, Switzerland

^8^ Center for Integrative Human Physiology (ZIHP), University of Zurich, Zurich, Switzerland

^9^ University Research Priority Program (URPP), Dynamic of Healthy Aging, University of Zurich, Zurich, Switzerland

^10^ Department of Special Education, King Abdulaziz University, Jeddah, Saudi Arabia

^#a^ Current address: Division Neuropsychology, Department of Psychology, University of Zurich, Zurich, Switzerland

*** Corresponding author**

Email: j.haenggi@psychologie.uzh.ch (J.H.)

**
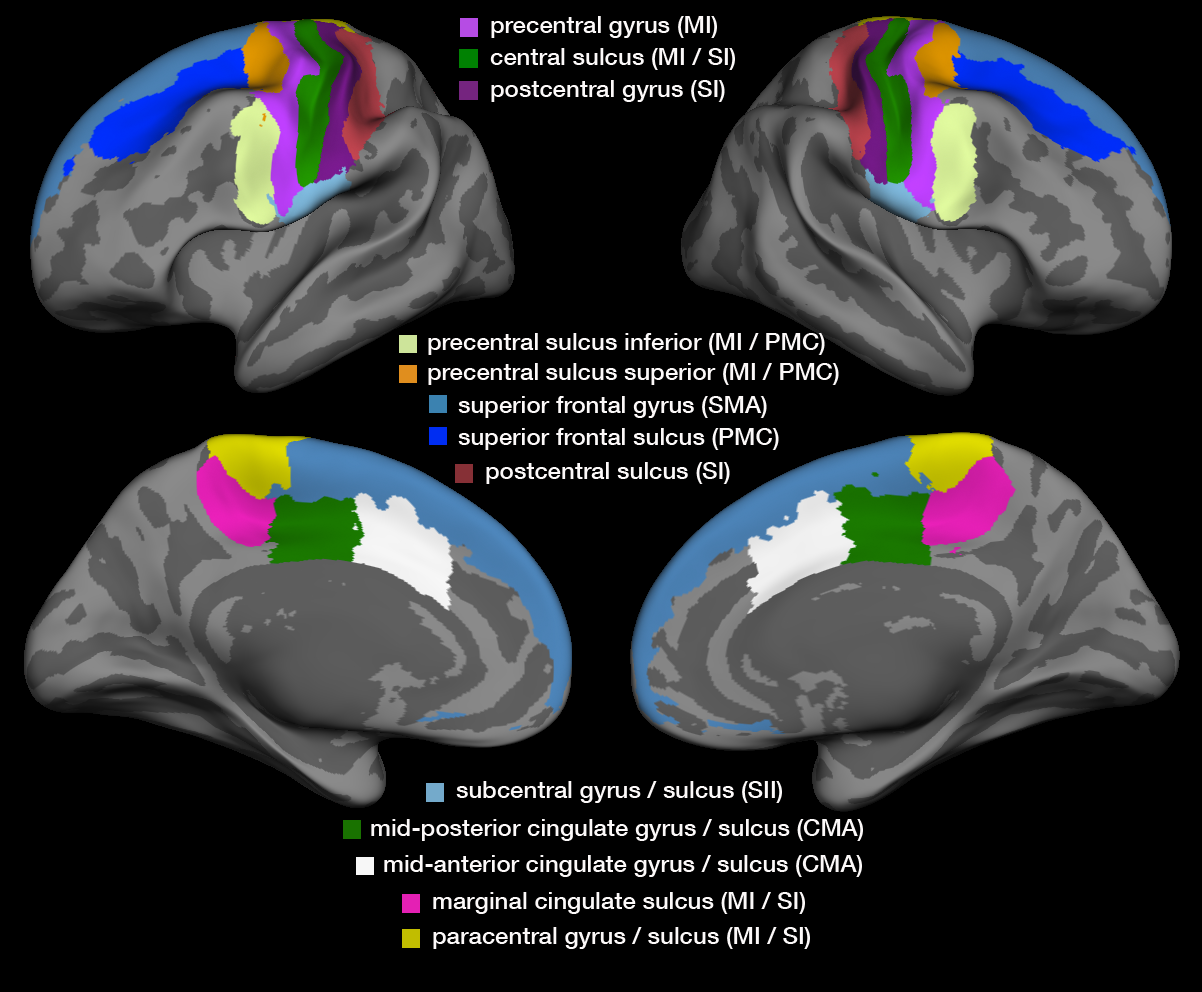
**

**S1 Fig. Regions of interest used in the surface-based morphometric analysis.** The clusters found in the voxel-based morphometric analysis are located within these regions of interest (ROIs). The exact boundaries of these ROIs can be found elsewhere [[1](#_ENREF_1)]. The left column shows the left hemisphere and the right column shows the right hemisphere. The first row represents lateral views of mean inflated surface models derived from the subjects under investigation and rotated by 30° in order to have a better view into the central sulcus. The second row represents the medial views. The functional brain areas located within these ROIs are reported in brackets. Abbreviations: CMA, cingulate motor area; MI, primary motor cortex; PMC, premotor cortex; SI, primary somatosensory cortex; SII, secondary somatosensory cortex; SMA, supplementary motor area.

**Supplementary references**

1. Destrieux C, Fischl B, Dale A, Halgren E (2010) Automatic parcellation of human cortical gyri and sulci using standard anatomical nomenclature. Neuroimage 53: 1-15.
