# Supplementary material for: Structural Brain Correlates Associated with Professional Handball Playing
Source: PLoS One. 2015 Apr 27;10(4):e0124222. doi: 10.1371/journal.pone.0124222 (PMC4411074; doi:10.1371/journal.pone.0124222)
Supplement: S2 Fig — The surface-based morphometric approach also revealed increased cortical volume in professional handball players compared with control participants in identical, similar and other regions as found in the voxel-based morphometric analysis. The statistical parametric maps were height-thresholded at p < 0.05 (uncorrected for multiple comparisons) as well as cluster extent-thresholded by considering only clusters larger than 25 mm2. The left column shows the left hemisphere and the right column shows the right hemisphere. The first row represents lateral views of mean inflated surface models derived from the subjects under investigation and rotated by 30° in order to have a better view into the central sulcus. The second row represents the medial views. More detailed information of the clusters presented can be found in S1 Table. Abbreviations: CMA, cingulate motor area; MI, primary motor cortex; PMC, premotor cortex; SI, primary somatosensory cortex; SMA, supplementary motor area. (DOCX) [file pone.0124222.s002.docx]

**Structural Brain Correlates Associated with Professional Handball Playing**

Jürgen Hänggi^1*,#a^, Nicolas Langer^1-3^, Kai Lutz^1,4,5^, Karin Birrer^1,6^, Susan Mérillat^1,7^ and Lutz Jäncke^1,7-10^

^1^ Division Neuropsychology, Department of Psychology, University of Zurich, Zurich, Switzerland

^2^ Neural Systems Lab, The City College of New York, New York, NY, USA

^3^ Child Mind Institute, New York, NY, USA

^4^ Center for Neurology and Rehabilitation, cereneo AG, Vitznau, Switzerland

^5^ Department of Neurology, University Hospital Zurich, Zurich, Switzerland

^6^ Rehabilitation Center Affoltern am Albis, University Children’s Hospital Zurich, Affoltern am Albis, Switzerland

^7^ International Normal Aging and Plasticity Imaging Center (INAPIC), University of Zurich, Zurich, Switzerland

^8^ Center for Integrative Human Physiology (ZIHP), University of Zurich, Zurich, Switzerland

^9^ University Research Priority Program (URPP), Dynamic of Healthy Aging, University of Zurich, Zurich, Switzerland

^10^ Department of Special Education, King Abdulaziz University, Jeddah, Saudi Arabia

^#a^ Current address: Division Neuropsychology, Department of Psychology, University of Zurich, Zurich, Switzerland

*** Corresponding author**

Email: j.haenggi@psychologie.uzh.ch (J.H.)

**
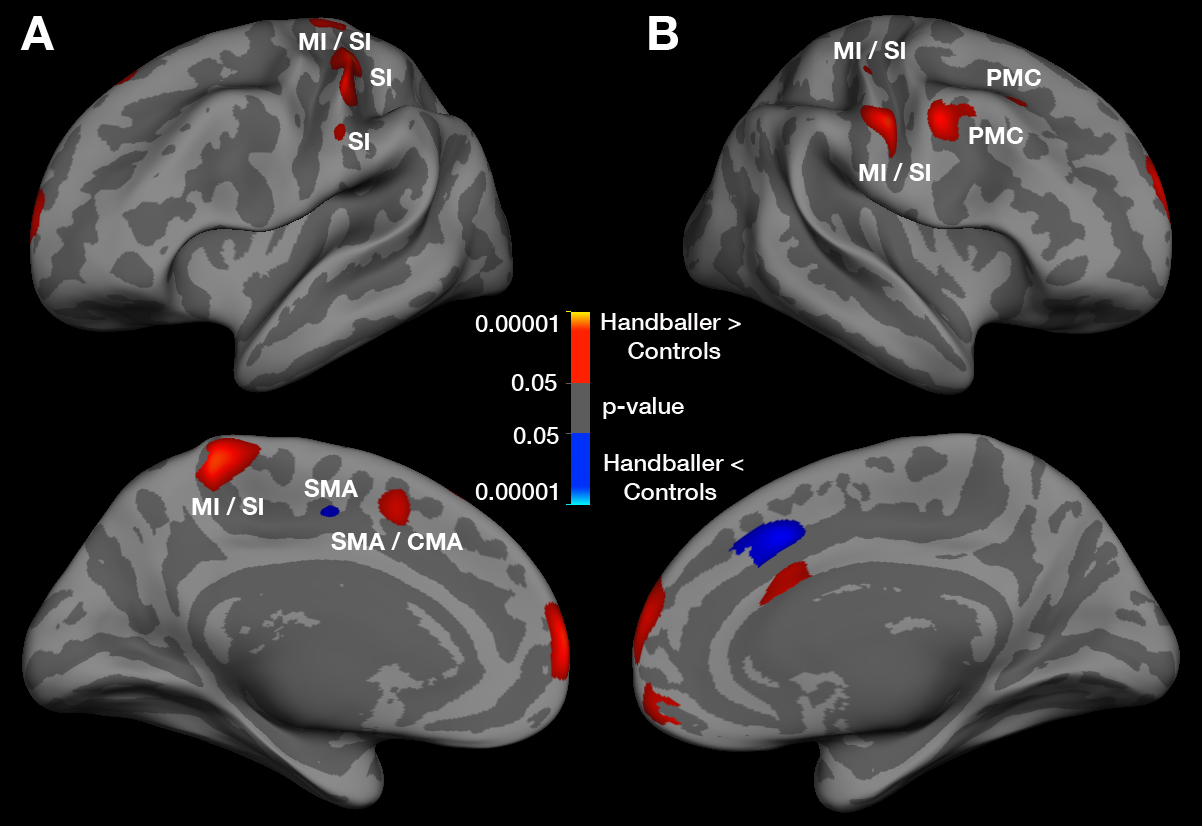
**

**S2 Fig. Replication and extension of the voxel-based morphometric finding reported in the main manuscript.** The surface-based morphometric approach also revealed increased cortical volume in professional handball players compared with control participants in identical, similar and other regions as found in the voxel-based morphometric analysis. The statistical parametric maps were height-thresholded at p < 0.05 (uncorrected for multiple comparisons) as well as cluster extent-thresholded by considering only clusters larger than 25 mm^2^. The left column shows the left hemisphere and the right column shows the right hemisphere. The first row represents lateral views of mean inflated surface models derived from the subjects under investigation and rotated by 30° in order to have a better view into the central sulcus. The second row represents the medial views. More detailed information of the clusters presented can be found in Supplementary Table S1. Abbreviations: CMA, cingulate motor area; MI, primary motor cortex; PMC, premotor cortex; SI, primary somatosensory cortex; SMA, supplementary motor area.
