# Supplementary material for: Structural Brain Correlates Associated with Professional Handball Playing
Source: PLoS One. 2015 Apr 27;10(4):e0124222. doi: 10.1371/journal.pone.0124222 (PMC4411074; doi:10.1371/journal.pone.0124222)
Supplement: S4 Fig — The statistical parametric maps were height-thresholded at p < 0.05 (uncorrected for multiple comparisons) as well as cluster extent thresholded by considering only clusters larger than 25 mm2. A) Left hemisphere and B) right right hemisphere. The first row represents lateral views of mean inflated surface models derived from the subjects under investigation and rotated by 30° in order to have a better view into the central sulcus. The second row represents the medial views. The inset in A) represent the PMC cluster rotated down by 60° to reveal that this is one connected cluster. The inset in B) represent the SII cluster rotated up by 60° to reveal the whole extent of this cluster. More detailed information of the clusters presented can be found in S1 Table. Abbreviations: CMA, cingulate motor area; MI, primary motor cortex; PMC, premotor cortex; SI, primary somatosensory cortex; SII, secondary somatosensory cortex; SMA, supplementary motor area. (DOCX) [file pone.0124222.s004.docx]

**Structural Brain Correlates Associated with Professional Handball Playing**

Jürgen Hänggi^1*,#a^, Nicolas Langer^1-3^, Kai Lutz^1,4,5^, Karin Birrer^1,6^, Susan Mérillat^1,7^ and Lutz Jäncke^1,7-10^

^1^ Division Neuropsychology, Department of Psychology, University of Zurich, Zurich, Switzerland

^2^ Neural Systems Lab, The City College of New York, New York, NY, USA

^3^ Child Mind Institute, New York, NY, USA

^4^ Center for Neurology and Rehabilitation, cereneo AG, Vitznau, Switzerland

^5^ Department of Neurology, University Hospital Zurich, Zurich, Switzerland

^6^ Rehabilitation Center Affoltern am Albis, University Children’s Hospital Zurich, Affoltern am Albis, Switzerland

^7^ International Normal Aging and Plasticity Imaging Center (INAPIC), University of Zurich, Zurich, Switzerland

^8^ Center for Integrative Human Physiology (ZIHP), University of Zurich, Zurich, Switzerland

^9^ University Research Priority Program (URPP), Dynamic of Healthy Aging, University of Zurich, Zurich, Switzerland

^10^ Department of Special Education, King Abdulaziz University, Jeddah, Saudi Arabia

^#a^ Current address: Division Neuropsychology, Department of Psychology, University of Zurich, Zurich, Switzerland

*** Corresponding author**

Email: j.haenggi@psychologie.uzh.ch (J.H.)

**
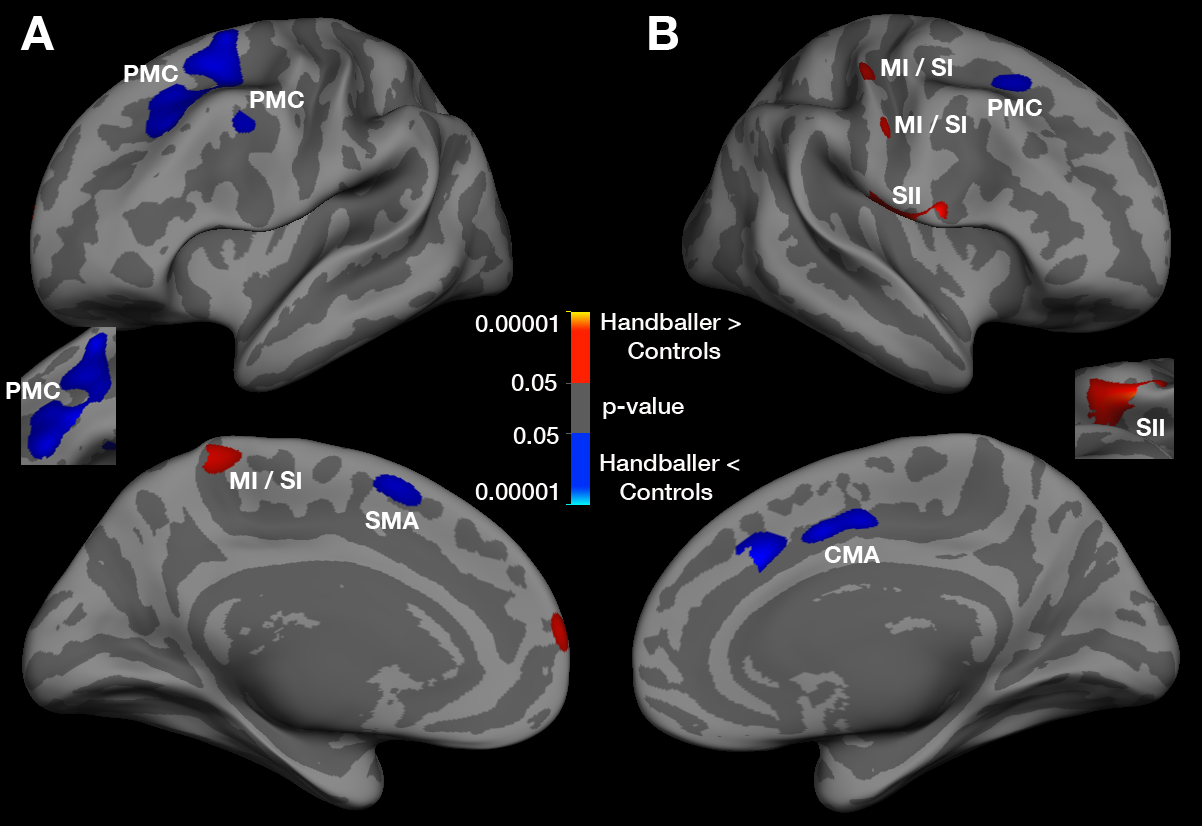
**

**S4 Fig. None, only marginal or inverse differences in cortical thickness in brain regions that showed increased cortical volume in professional handball players.** The statistical parametric maps were height-thresholded at p < 0.05 (uncorrected for multiple comparisons) as well as cluster extent thresholded by considering only clusters larger than 25 mm^2^. A) Left hemisphere and B) right right hemisphere. The first row represents lateral views of mean inflated surface models derived from the subjects under investigation and rotated by 30° in order to have a better view into the central sulcus. The second row represents the medial views. The inset in A) represent the PMC cluster rotated down by 60° to reveal that this is one connected cluster. The inset in B) represent the SII cluster rotated up by 60° to reveal the whole extent of this cluster. More detailed information of the clusters presented can be found in Supplementary Table S1. Abbreviations: CMA, cingulate motor area; MI, primary motor cortex; PMC, premotor cortex; SI, primary somatosensory cortex; SII, secondary somatosensory cortex; SMA, supplementary motor area.
