# Supplementary material for: Structural Brain Correlates Associated with Professional Handball Playing
Source: PLoS One. 2015 Apr 27;10(4):e0124222. doi: 10.1371/journal.pone.0124222 (PMC4411074; doi:10.1371/journal.pone.0124222)
Supplement: S1 Methods — The clusters found in the voxel-based morphometric analysis are located within these regions of interest (ROIs). The exact boundaries of these ROIs can be found elsewhere (see supplementary reference #13 in S1 Methods). The left column shows the left hemisphere and the right column shows the right hemisphere. The first row represents lateral views of mean inflated surface models derived from the subjects under investigation and rotated by 30° in order to have a better view into the central sulcus. The second row represents the medial views. The functional brain areas located within these ROIs are reported in brackets. Abbreviations: CMA, cingulate motor area; MI, primary motor cortex; PMC, premotor cortex; SI, primary somatosensory cortex; SII, secondary somatosensory cortex; SMA, supplementary motor area. (DOCX) [file pone.0124222.s005.docx]

**Structural Brain Correlates Associated with Professional Handball Playing**

Jürgen Hänggi^1*,#a^, Nicolas Langer^1-3^, Kai Lutz^1,4,5^, Karin Birrer^1,6^, Susan Mérillat^1,7^ and Lutz Jäncke^1,7-10^

^1^ Division Neuropsychology, Department of Psychology, University of Zurich, Zurich, Switzerland

^2^ Neural Systems Lab, The City College of New York, New York, NY, USA

^3^ Child Mind Institute, New York, NY, USA

^4^ Center for Neurology and Rehabilitation, cereneo AG, Vitznau, Switzerland

^5^ Department of Neurology, University Hospital Zurich, Zurich, Switzerland

^6^ Rehabilitation Center Affoltern am Albis, University Children’s Hospital Zurich, Affoltern am Albis, Switzerland

^7^ International Normal Aging and Plasticity Imaging Center (INAPIC), University of Zurich, Zurich, Switzerland

^8^ Center for Integrative Human Physiology (ZIHP), University of Zurich, Zurich, Switzerland

^9^ University Research Priority Program (URPP), Dynamic of Healthy Aging, University of Zurich, Zurich, Switzerland

^10^ Department of Special Education, King Abdulaziz University, Jeddah, Saudi Arabia

^#a^ Current address: Division Neuropsychology, Department of Psychology, University of Zurich, Zurich, Switzerland

*** Corresponding author**

Email: j.haenggi@psychologie.uzh.ch (J.H.)

**Supplementary methods**

Cortical surface reconstruction, cortical parcellation as well as subcortical volumetric segmentation were performed with the FreeSurfer image analysis suite (version 5.3.0), which is documented and freely available online (<http://surfer.nmr.mgh.harvard.edu/>). The technical details of these procedures are described in prior publications [[1-8](#_ENREF_1)]. The 3D structural T1-weighted MRI scan was used to construct models of each subject’s cortical surface in order to measure cortical thickness and cortical surface area. This fully automated procedure comprised segmentation of the cortical white matter [[8](#_ENREF_8)], tessellation of the grey/white matter junction, inflation of the folded surface tessellation patterns [[5](#_ENREF_5),[6](#_ENREF_6)] and automatic correction of topological defects in the resulting manifold [[2](#_ENREF_2)]. This surface was then used as starting point for a deformable surface algorithm designed to find the grey / white and pial (grey matter / cerebrospinal fluid) interfaces with sub-millimetre precision [[1](#_ENREF_1)]. The procedures for measuring cortical thickness have been validated against histological analysis [[9](#_ENREF_9)] and manual measurements [[10](#_ENREF_10),[11](#_ENREF_11)]. This method uses both intensity and continuity information from the surfaces in the deformation procedure in order to interpolate surface locations for regions in which the MRI scan is ambiguous [[1](#_ENREF_1)]. For each subject, cortical thickness of the cortical ribbon was computed on a uniform grid (comprised by vertices) with 1 mm spacing across both cortical hemispheres, with the thickness being defined by the shortest distance between the grey/white and pial surface models. The thickness maps produced are not limited to the voxel resolution of the image and thus sensitive for sub-millimetre differences between groups [[1](#_ENREF_1)]. The way in which the resolution of the cortical thickness maps goes beyond the resolution of the original acquisition is conceptually similar to a (conventional) partial volume correction procedure. The cortex is smooth at the spatial scale of a several millimetres, which is imposed as constraint by FreeSurfer to estimate the location of the surface with subvoxel accuracy. For instance, if a given voxel is darker than its neighbouring grey matter it probably contains more cerebrospinal fluid and so the surface model is at a slightly different position than if the neighbouring voxels were brighter and therefore contain probably more white matter. Cortical thickness, surface area, and volume measures were mapped to the inflated surface of each participant’s brain reconstruction, this allowing visualization of data across the entire cortical surface (gyri and sulci) without the data being obscured by cortical folding. Data were re-sampled for all subjects and rendered onto a common spherical coordinate system [[6](#_ENREF_6)]. Then a surface-based vertex-wise cortical thickness, surface area, and volume map were computed for each participant. For the whole-brain vertex-wise analysis, the data were smoothed on the surface tessellation using an iterative nearest- neighbour averaging procedure with 166 iterations on the left hemisphere and 167 iterations on the right hemisphere, corresponding to a 2D surface-based diffusion smoothing kernel with a full width at half maximum of 15 mm.

In addition, the cerebral cortex was parcellated into units based on gyral/sulcal structure as implemented in FreeSurfer [[7](#_ENREF_7),[12](#_ENREF_12),[13](#_ENREF_13)]. Some of these parcellations (see S1 Fig.) served as regions of interest (ROIs) to restrict the statistical analysis to brain structures that are part of the somatosensory-motor network.

**Supplementary references**

1. Fischl B, Dale AM (2000) Measuring the thickness of the human cerebral cortex from magnetic resonance images. Proceedings of the National Academy of Sciences of the United States of America 97: 11050-11055.

2. Fischl B, Liu A, Dale AM (2001) Automated manifold surgery: constructing geometrically accurate and topologically correct models of the human cerebral cortex. Medical Imaging, IEEE Transactions on 20: 70-80.

3. Fischl B, Salat DH, Busa E, Albert M, Dieterich M, Haselgrove C, et al. (2002) Whole Brain Segmentation: Automated Labeling of Neuroanatomical Structures in the Human Brain. Neuron 33: 341-355.

4. Fischl B, Salat DH, van der Kouwe AJW, Makris N, Ségonne F, Quinn BT, et al. (2004) Sequence-independent segmentation of magnetic resonance images. NeuroImage 23: S69-S84.

5. Fischl B, Sereno MI, Dale AM (1999) Cortical Surface-Based Analysis: II: Inflation, Flattening, and a Surface-Based Coordinate System. NeuroImage 9: 195-207.

6. Fischl B, Sereno MI, Tootell RBH, Dale AM (1999) High-resolution intersubject averaging and a coordinate system for the cortical surface. Human Brain Mapping 8: 272-284.

7. Fischl B, van der Kouwe A, Destrieux C, Halgren E, Ségonne F, Salat DH, et al. (2004) Automatically Parcellating the Human Cerebral Cortex. Cereb Cortex 14: 11-22.

8. Dale AM, Fischl B, Sereno MI (1999) Cortical Surface-Based Analysis: I. Segmentation and Surface Reconstruction. NeuroImage 9: 179-194.

9. Rosas HD, Liu AK, Hersch S, Glessner M, Ferrante RJ, Salat DH, et al. (2002) Regional and progressive thinning of the cortical ribbon in Huntington's disease. Neurology 58: 695-701.

10. Kuperberg GR, Broome MR, McGuire PK, David AS, Eddy M, Ozawa F, et al. (2003) Regionally Localized Thinning of the Cerebral Cortex in Schizophrenia. Arch Gen Psychiatry 60: 878-888.

11. Salat DH, Buckner RL, Snyder AZ, Greve DN, Desikan RSR, Busa E, et al. (2004) Thinning of the Cerebral Cortex in Aging. Cereb Cortex 14: 721-730.

12. Desikan RS, Ségonne F, Fischl B, Quinn BT, Dickerson BC, Blacker D, et al. (2006) An automated labeling system for subdividing the human cerebral cortex on MRI scans into gyral based regions of interest. NeuroImage 31: 968-980.

13. Destrieux C, Fischl B, Dale A, Halgren E (2010) Automatic parcellation of human cortical gyri and sulci using standard anatomical nomenclature. NeuroImage 53: 1-15.
