# Supplementary material for: Structural Brain Correlates Associated with Professional Handball Playing
Source: PLoS One. 2015 Apr 27;10(4):e0124222. doi: 10.1371/journal.pone.0124222 (PMC4411074; doi:10.1371/journal.pone.0124222)
Supplement: S1 Results — Additional findings that were statistically not significant, but showed a trend toward significance (0.05 < p < 0.10). (DOCX) [file pone.0124222.s006.docx]

**Structural Brain Correlates Associated with Professional Handball Playing**

Jürgen Hänggi^1*,#a^, Nicolas Langer^1-3^, Kai Lutz^1,4,5^, Karin Birrer^1,6^, Susan Mérillat^1,7^ and Lutz Jäncke^1,7-10^

^1^ Division Neuropsychology, Department of Psychology, University of Zurich, Zurich, Switzerland

^2^ Neural Systems Lab, The City College of New York, New York, NY, USA

^3^ Child Mind Institute, New York, NY, USA

^4^ Center for Neurology and Rehabilitation, cereneo AG, Vitznau, Switzerland

^5^ Department of Neurology, University Hospital Zurich, Zurich, Switzerland

^6^ Rehabilitation Center Affoltern am Albis, University Children’s Hospital Zurich, Affoltern am Albis, Switzerland

^7^ International Normal Aging and Plasticity Imaging Center (INAPIC), University of Zurich, Zurich, Switzerland

^8^ Center for Integrative Human Physiology (ZIHP), University of Zurich, Zurich, Switzerland

^9^ University Research Priority Program (URPP), Dynamic of Healthy Aging, University of Zurich, Zurich, Switzerland

^10^ Department of Special Education, King Abdulaziz University, Jeddah, Saudi Arabia

^#a^ Current address: Division Neuropsychology, Department of Psychology, University of Zurich, Zurich, Switzerland

*** Corresponding author**

Email: j.haenggi@psychologie.uzh.ch (J.H.)

**Supplementary results**

**Voxel-based morphometry**

When increasing the statistical threshold (p < 0.10, corrected) for explorative purposes and due to the small sample size associated with reduced statistical power, one additional cluster with volumetric GM differences between handball players and control women can be found. This cluster is located in the vermis of the cerebellum (MNI coordinates: x = -2, y = -52, z = -40), but showed reduced instead of increased GM volume in handball players (not shown).

There were no significant correlations between age of handball training commencement or years of handball training and local GM volume at a statistical threshold of p < 0.05, corrected for multiple comparisons. However, when increasing the statistical threshold (p < 0.10, corrected) a small cluster with a negative correlation between age of handball training commencement and GM volume was evident in the left MI/SI hand representation (MNI coordinates: x = -46, y = -16, z = 40) located in the central sulcus (not shown).

**Diffusion tensor imaging**

Within the cluster that showed significant differences in FA as well as axial diffusivity (see Figs. 2A and 2C in the main manuscript) there was a statistical trend (0.05 < p < 0.10, corrected) toward decreased radial diffusivity in handball players compared with control women.

Years of handball training experience were inversely associated with radial diffusivity in a cluster located in the right CST on the height of the corpus callosum (see Fig. 2D and Table 4 in the main manuscript). This cluster also showed positive correlations between years of handball training experience and FA, but only on a trend level. Also on a trend level, mean diffusivity was inversely related to years of handball training experience in a cluster located in the left CST on the height of the corpus callosum (not shown).

With respect to the corpus callosum and after correction for multiple comparisons, there was no significant cluster that showed differences between groups in FA, mean, axial, or radial diffusivity. However, when increasing the statistical threshold (p < 0.06, corrected), one additional small cluster with increased axial diffusivity in handball players compared with control women can be found in the splenium of the corpus callosum (MNI coordinates: x = -11, y = -36, z = 23; not shown). No significant differences between the groups were found in any of the diffusivity parameters within the cingulum, which served as a negative control tract.

There were no significant correlations between age of handball training commencement or years of handball training and the DTI parameters. However, when increasing the statistical threshold (p < 0.10, corrected) two interesting clusters emerged. Years of handball training positively correlated with FA and negatively correlated with mean diffusivity in a cluster located in the right CST (MNI coordinates: x = 24, y = -14, z = 37). Years of handball training, however, negatively correlated with mean and radial diffusivity in a cluster located in the left CST (MNI coordinates: x = -22, y = -21, z = 37; not shown).

**Subcortical structures**

A multivariate analysis of covariance correcting for total GM volume revealed significantly increased volumes of all basal ganglia structures (putamen, caudate nucleus, pallidum) and the thalami together (multivariate) in professional handball players compared with control women (see main manuscript). Subsequent post hoc univariate analyses of covariance (ANCOVA) correcting for total GM volume revealed only a statistical trend toward a larger right caudate nucleus volume in handball players compared with control women (F_(1,20)_ = 3.88; p = 0.063; η_p_^2^ = 0.16), although all structures were larger in handball players (S2 Table).

Age of training commencement was inversely related to the volume of the left caudate nucleus (r = -0.58, p = 0.062) and positively related to the volume of the left putamen (r = 0.52, p = 0.10), but only on a trend level towards statistical significance (uncorrected for multiple comparisons). These effects might be confounded by age. Across both groups, age was inversely associated with the volume of the left caudate nucleus (r = -0.26), but was statistically not significant (p = 0.24, two-tailed), and not associated with the volume of the left putamen at all (r = 0.03, p = 0.90). However, when age was correlated within each group separately, the resulting correlations differ considerably between groups. Within the handball players, age was inversely associated with the volume of the left caudate nucleus (r = -0.40) and positively associated with the volume of the left putamen (r = 0.47), but these correlations did not reach statistical significance (p = 0.22 and p = 0.14, respectively). Within the control women, age was neither associated with the volume of the left caudate nucleus (r = -0.01, p = 0.97) nor with that of the left putamen (r = 0.08, p = 0.80). The differences in the associations between age and volume of the left caudate nucleus and left putamen might suggest that there is an effect on theses volumes in handball players that goes beyond the common age effect, which however was absent in the control women investigated in the present study most probably due to the narrow age range (20-30 years).

Although the differences in correlation magnitude are considerably, when comparing these correlation coefficients between groups using Fisher’s r-to-z-transformation neither the correlation difference of the caudate (z = -0.85, p = 0.20, one-tailed) nor that of the putamen (z = 0.88, p = 0.19, one-tailed) were statistically significantly different between groups.

**Regional corpus callosum volumes**

Subsequent post hoc ANCOVAs corrected for total GM volume revealed that the posterior and the mid-posterior region of the corpus callosum are larger in volume in handball players compared with control women, but only on a trend level towards statistical significance (posterior: F_(1,20)_ = 3.03; p = 0.097; η_p_^2^ = 0.13; mid-posterior: F_(1,20)_ = 3.97; p = 0.060; η_p_^2^ = 0.17). The sum of all corpus callosum regional volumes tented to be larger in handball players compared with control women (F_(1,20)_ = 3.44; p = 0.079; η_p_^2^ = 0.15) (S2 Table).

Years of handball training were positively related to the volume of the mid-posterior portion of the corpus callosum (r = 0.55, p = 0.080), but only on a trend level towards statistical significance (uncorrected for multiple comparisons).

**Global cerebellar grey and white matter volumes**

With respect to the cerebellum, an ANCOVA corrected for total GM volume revealed trends towards larger cerebellar GM volumes in handball players (left hemisphere: F_(1,20)_ = 2.43; p = 0.14; η_p_^2^ = 0.11; right hemisphere: F_(1,20)_ = 3.02; p = 0.098; η_p_^2^ = 0.13), but these differences did not reach statistical significance.

Years of handball training were negatively related to the right cerebellar WM volume (r = -0.52, p = 0.099), but only on a trend level towards statistical significance (uncorrected for multiple comparisons).

**Associations between neuroplastic alterations**

The associations reported in the main manuscript were all positive and statistically significant. However, when using a more liberal statistical threshold (0.05 < p < 0.10, two-tailed), one inverse association was found. Mean GM probability of the VBM-derived clusters and cerebellar WM volume were negatively correlated (r = -0.357, p = 0.095). No further inverse associations were found between any of the measures reported in the main manuscript.

**Surface-based morphometry**

The aim of the surface-based morphometric analysis was twofold. On one hand we aimed at replicating the VBM findings with a different methodology (i.e. SBM) and on the other hand we aimed at determining whether the increased probabilistic cortical volume found in the VBM analysis is driven by increases in cortical thickness or cortical surface area or increases in both measures. The results of the SBM analysis are summarised in S1 Table and shown in S2-S4 Figs. In brief, we found regions with increased cortical volume in handball players compared with control women in the right and left hemispheric ROIs including clusters in the functional areas MI (BA 4a / 4p), SI (BA 3a / 3b / 1), premotor cortex (BA 6), and SMA / CMA (medial BA 4 / 6 / 24) (S1 Table and S2 Fig.). These cortical volume differences were driven by increases in cortical surface area, increases in cortical thickness or increases in both measures (S1 Table and S3 and S4 Figs., respectively). For example, the cluster with increased probabilistic grey matter volume found in the SMA / CMA region in the VBM analysis (MNI coordinates: x = -2, y = 8, z = 42, see Fig. 1A and Table 2) has also be revealed by the SBM analysis (x = -8, y = 13, z = 48, see SMA / CMA label in S2 Fig. and letter F in S1 Table). Subsequent analyses of cortical surface area and thickness revealed that the volume increase in this SMA / CMA cluster is solely driven by increases in cortical surface area (x = -9, y = 15, z = 54, see S3 Fig. and letter F in S2 Table) and not by cortical thickness (x = -9, y = 12, z = 58, see S4 Fig. and letter F in S2 Table). Moreover, cortical thickness in this region was reduced in handball players compared with control women. Similar patterns of morphological changes between handball players and control women were found in other clusters as well. No correlations between age of handball training commencement or years of handball training were computed for the surface-based morphometric measures.
