# Supplementary material for: Structural Brain Correlates Associated with Professional Handball Playing
Source: PLoS One. 2015 Apr 27;10(4):e0124222. doi: 10.1371/journal.pone.0124222 (PMC4411074; doi:10.1371/journal.pone.0124222)
Supplement: S1 Table — Clusters within the different measures are ordered by decreasing statistical significance. Bold printed clusters represent cluster within the predicted regions of interest (ROIs). The ROIs also contain parts of the brain for which we did not pose any hypothesis because these ROIs were chosen from a predefined atlas. However, the clusters found outside of the predicted regions but inside the ROIs are presented for completeness. (HABA > CON) indicates clusters that showed increased values in handball players compared with control women. (CON>HABA) indicates clusters that showed decreased values in handball players compared with control women. The statistical parametric maps reported here were height-thresholded at p < 0.05 (uncorrected for multiple comparisons) as well as cluster extent thresholded by considering only clusters larger than 25 mm2 in size. Clusters indicated by the paragraph sign (§) survived error correction for multiple comparisons by using Monte Carlo simulations on the cluster size using 5000 permutations. The ampersand sign (&) highlights clusters with cortical differences in handball players compared with controls as found by both the voxel-based as well as the surface-based morphometric analysis. (A) to (H) indicate corresponding clusters across cortical measures. Abbreviations: BA, Brodmann area; CMA; cingulate motor area; MI, primary motor area; MNI, Montreal neurological institute (space); PMC, premotor cortex; SI, primary somatosensory cortex; SII, secondary somatosensory cortex; SMA, supplementary motor area. (DOCX) [file pone.0124222.s007.docx]

**Structural Brain Correlates Associated with Professional Handball Playing**

Jürgen Hänggi^1*,#a^, Nicolas Langer^1-3^, Kai Lutz^1,4,5^, Karin Birrer^1,6^, Susan Mérillat^1,7^ and Lutz Jäncke^1,7-10^

^1^ Division Neuropsychology, Department of Psychology, University of Zurich, Zurich, Switzerland

^2^ Neural Systems Lab, The City College of New York, New York, NY, USA

^3^ Child Mind Institute, New York, NY, USA

^4^ Center for Neurology and Rehabilitation, cereneo AG, Vitznau, Switzerland

^5^ Department of Neurology, University Hospital Zurich, Zurich, Switzerland

^6^ Rehabilitation Center Affoltern am Albis, University Children’s Hospital Zurich, Affoltern am Albis, Switzerland

^7^ International Normal Aging and Plasticity Imaging Center (INAPIC), University of Zurich, Zurich, Switzerland

^8^ Center for Integrative Human Physiology (ZIHP), University of Zurich, Zurich, Switzerland

^9^ University Research Priority Program (URPP), Dynamic of Healthy Aging, University of Zurich, Zurich, Switzerland

^10^ Department of Special Education, King Abdulaziz University, Jeddah, Saudi Arabia

^#a^ Current address: Division Neuropsychology, Department of Psychology, University of Zurich, Zurich, Switzerland

*** Corresponding author**

Email: j.haenggi@psychologie.uzh.ch (J.H.

**S1 Table. Regions with increased or decreased cortical volume, surface area, and thickness in the somatosensory-motor network in handball players derived from the surface-based morphometric analysis.**

_______________________________________________________________________________________________________________________________________________

|  | **Direction** | **Cluster** | **Error** | **MNI coordinates** | | |
| --- | --- | --- | --- | --- | --- | --- |
| **Name (abbreviation)** | **of effect** | **size (mm^2^)** | **probability** | **x** | **y** | **z** |
| _______________________________________________________________________________________________________________________________________________ | | | | | | |
| *Right cortical volume* |  |  |  |  |  |  |
|  |  |  |  |  |  |  |
| **(A) Central sulcus (MI / SI, BA 4p / 3b)** | **HABA > CON** | **393.7** | **0.001** | **48.1** | **12.7** | **40.3** |
| Medial frontal pole § | HABA > CON | 496.1 | 0.002 | 9.8 | 65.9 | 22.0 |
| Paracingulate gyrus | CON > HABA | 256.8 | 0.003 | 7.9 | 20.1 | 42.3 |
| **(B) Precentral sulcus (PMC, BA 6)** | **HABA > CON** | **324.7** | **0.004** | **45.0** | **3.0** | **41.2** |
| Medial orbitofrontal cortex | HABA > CON | 186.8 | 0.010 | 9.2 | 52.9 | -11.8 |
| Anterior cingulate gyrus | HABA > CON | 138.5 | 0.011 | 6.2 | 19.5 | 25.9 |
| **(C) Superior frontal sulcus (PMC, BA 6)** | **HABA > CON** | **55.3** | **0.022** | **28.6** | **18.8** | **44.6** |
|  |  |  |  |  |  |  |
| **(D) Central sulcus (MI / SI, BA 4p / 3b)** | **HABA > CON** | **33.3** | **0.045** | **42.3** | **23.2** | **61.7** |
|  |  |  |  |  |  |  |
| *Right mid-cortical surface area* |  |  |  |  |  |  |
|  |  |  |  |  |  |  |
| **(A) Central sulcus (MI / SI, BA 4p / 3b)** | **HABA > CON** | **257.1** | **0.001** | **51.3** | **12.4** | **45.5** |
| Medial frontal pole § | HABA > CON | 563.0 | 0.003 | 8.3 | 64.2 | 14.1 |
| **(C) Superior frontal sulcus (PMC, BA 6)** | **HABA > CON** | **162.3** | **0.012** | **28.6** | **15.3** | **45.2** |
| Cingulate gyrus | HABA > CON | 248.3 | 0.018 | 8.0 | 15.4 | 33.6 |
| Paracingulate gyrus | CON > HABA | 46.5 | 0.026 | 8.1 | 19.5 | 42.5 |
| **(B) Precentral sulcus (PMC, BA 6)** | **HABA > CON** | **32.9** | **0.001** | **45.7** | **4.7** | **46.7** |
|  |  |  |  |  |  |  |
| *Right cortical thickness* |  |  |  |  |  |  |
| **Central operculum (SII, BA 43) §** | **HABA > CON** | **434.2** | **0.0005** | **54.1** | **0.7** | **9.6** |
| Paracingulate gyrus | CON > HABA | 167.5 | 0.002 | 10.4 | 29.2 | 34.2 |
| **Mid-anterior cingulate gyrus (CMA)** | **CON > HABA** | **175.6** | **0.009** | **13.0** | **7.5** | **42.5** |
|  |  |  |  |  |  |  |
| **(D) Central sulcus (MI / SI, BA 4p / 3b)** | **HABA > CON** | **147.5** | **0.016** | **43.4** | **21.8** | **61.3** |
| **Superior frontal gyrus (PMC, BA 6)** | **CON > HABA** | **125.6** | **0.018** | **22.1** | **14.9** | **50.2** |
| **(A) Central sulcus (MI / SI, BA 4p / 3b)** | **HABA > CON** | **58.5** | **0.023** | **51.1** | **-10.8** | **39.5** |
|  |  |  |  |  |  |  |
| *Left cortical volume* |  |  |  |  |  |  |
|  |  |  |  |  |  |  |
| **Postcentral gyrus (SI, BA 1) §** | **HABA > CON** | **752.8** | **0.001** | **-38.4** | **34.6** | **64.4** |
| **(E) Paracentral lobule (MI / SI, BA 4a / 3b)** | **HABA > CON** | **378.0** | **0.001** | **-13.9** | **-39.1** | **63.3** |
| Frontal pole | HABA > CON | 469.1 | 0.002 | -9.0 | 66.3 | 11.6 |
| **(F) Medial superior frontal cortex (SMA / CMA) &** | **HABA > CON** | **118.4** | **0.010** | **-8.1** | **13.1** | **48.3** |
|  |  |  |  |  |  |  |
| **(G) Central sulcus (MI / SI, BA 4a / 3b)** | **HABA > CON** | **133.1** | **0.016** | **-15.4** | **31.0** | **75.3** |
| **Medial superior frontal cortex (SMA)** | **CON > HABA** | **36.3** | **0.023** | **-11.7** | **-7.4** | **50.1** |
|  |  |  |  |  |  |  |
| **(H) Postcentral gyrus (SI, BA 3b)** | **HABA > CON** | **56.1** | **0.031** | **-55.9** | **16.8** | **41.0** |
| Superior frontal gyrus | HABA > CON | 49.6 | 0.035 | -10.9 | 31.7 | 55.4 |
|  |  |  |  |  |  |  |
| *Left mid-cortical surface area* |  |  |  |  |  |  |
| **(F) Medial superior frontal cortex (SMA / CMA)** | **HABA > CON** | **410.4** | **0.0004** | **-8.9** | **14.8** | **54.2** |
|  |  |  |  |  |  |  |
| **Postcentral gyrus (SI, BA 1) §** | **HABA > CON** | **855.2** | **0.001** | **-50.7** | **21.7** | **55.3** |
|  |  |  |  |  |  |  |
| **(H) Postcentral gyrus (SI, BA 3b)** | **HABA > CON** | **138.3** | **0.004** | **-55.1** | **13.4** | **43.8** |
|  |  |  |  |  |  |  |
| **(E) Paracentral lobule (MI / SI, BA 4a / 3b)** | **HABA > CON** | **349.5** | **0.013** | **-8.4** | **39.0** | **65.3** |
| Posterior cingulate gyrus | HABA > CON | 46.9 | 0.013 | -6.5 | -36.5 | 43.6 |
|  |  |  |  |  |  |  |
| **(G) Central sulcus (MI / SI, BA 4a / 3b)** | **HABA > CON** | **45.9** | **0.030** | **-18.2** | **36.6** | **67.2** |
| Frontal pole | HABA > CON | 90.7 | 0.039 | -9.0 | 66.8 | 9.8 |
| Middle cingulate gyrus | HABA > CON | 32.4 | 0.039 | -3.9 | 9.4 | 33.6 |
| Superior frontal sulcus | HABA > CON | 36.0 | 0.039 | -23.4 | 25.4 | 36.9 |
|  |  |  |  |  |  |  |
| *Left cortical thickness* |  |  |  |  |  |  |
| **Middle / superior frontal gyrus (PMC, BA 6) §** | **CON > HABA** | **1139.5** | **0.004** | **-28.2** | **25.4** | **40.9** |
|  |  |  |  |  |  |  |
| **(E) Paracentral lobule (MI / SI, BA 4a / 3b)** | **HABA > CON** | **141.2** | **0.011** | **-14.8** | **41.4** | **65.0** |
| Frontal pole | HABA > CON | 132.4 | 0.016 | -8.4 | 65.5 | 13.7 |
| **(F) Medial superior frontal cortex (SMA, BA 6)** | **CON > HABA** | **147.8** | **0.016** | **-9.1** | **12.4** | **57.8** |
| **Precentral sulcus (PMC; BA 6)** | **CON > HABA** | **84.1** | **0.025** | **-37.2** | **6.0** | **40.0** |

_______________________________________________________________________________________________________________________________________________

Clusters within the different measures are ordered by decreasing statistical significance. Bold printed clusters represent cluster within the predicted regions of interest (ROIs). The ROIs also contain parts of the brain for which we did not pose any hypothesis because these ROIs were chosen from a predefined atlas. However, the clusters found outside of the predicted regions but inside the ROIs are presented for completeness. (HABA > CON) indicates clusters that showed increased values in handball players compared with control women. (CON>HABA) indicates clusters that showed decreased values in handball players compared with control women. The statistical parametric maps reported here were height-thresholded at p < 0.05 (uncorrected for multiple comparisons) as well as cluster extent thresholded by considering only clusters larger than 25 mm^2^ in size. Clusters indicated by the paragraph sign (§) survived error correction for multiple comparisons by using Monte Carlo simulations on the cluster size using 5000 permutations. The ampersand sign (&) highlights clusters with cortical differences in handball players compared with controls as found by both the voxel-based as well as the surface-based morphometric analysis. (A) to (H) indicate corresponding clusters across cortical measures. Abbreviations: BA, Brodmann area; CMA; cingulate motor area; MI, primary motor area; MNI, Montreal neurological institute (space); PMC, premotor cortex; SI, primary somatosensory cortex; SII, secondary somatosensory cortex; SMA, supplementary motor area.
