# Supplementary material for: Structural Brain Correlates Associated with Professional Handball Playing
Source: PLoS One. 2015 Apr 27;10(4):e0124222. doi: 10.1371/journal.pone.0124222 (PMC4411074; doi:10.1371/journal.pone.0124222)
Supplement: S2 Table — *, corpus callosum volume was computed based on a mid-sagittal slice of 5 mm thickness. (DOCX) [file pone.0124222.s008.docx]

**Structural Brain Correlates Associated with Professional Handball Playing**

Jürgen Hänggi^1*,#a^, Nicolas Langer^1-3^, Kai Lutz^1,4,5^, Karin Birrer^1,6^, Susan Mérillat^1,7^ and Lutz Jäncke^1,7-10^

^1^ Division Neuropsychology, Department of Psychology, University of Zurich, Zurich, Switzerland

^2^ Neural Systems Lab, The City College of New York, New York, NY, USA

^3^ Child Mind Institute, New York, NY, USA

^4^ Center for Neurology and Rehabilitation, cereneo AG, Vitznau, Switzerland

^5^ Department of Neurology, University Hospital Zurich, Zurich, Switzerland

^6^ Rehabilitation Center Affoltern am Albis, University Children’s Hospital Zurich, Affoltern am Albis, Switzerland

^7^ International Normal Aging and Plasticity Imaging Center (INAPIC), University of Zurich, Zurich, Switzerland

^8^ Center for Integrative Human Physiology (ZIHP), University of Zurich, Zurich, Switzerland

^9^ University Research Priority Program (URPP), Dynamic of Healthy Aging, University of Zurich, Zurich, Switzerland

^10^ Department of Special Education, King Abdulaziz University, Jeddah, Saudi Arabia

^#a^ Current address: Division Neuropsychology, Department of Psychology, University of Zurich, Zurich, Switzerland

*** Corresponding author**

Email: j.haenggi@psychologie.uzh.ch (J.H.)

**S2 Table. Volumes of subcortical structures including the corpus callosum and cerebellum derived from the volumetric segmentation analysis.**

|  | **Handball players (n = 11)** | | | | **Control women (n = 12)** | | | | **Significance** |
| --- | --- | --- | --- | --- | --- | --- | --- | --- | --- |
| **Volume (cm^3^)** | **Mean** | **SD** | **Min.** | **Max.** | **Mean** | **SD** | **Min.** | **Max.** | **p-value** |
| **Basal ganglia and thalamus** |  |  |  |  |  |  |  |  |  |
| Left putamen | 6.55 | 0.30 | 5.94 | 6.94 | 6.09 | 0.92 | 4.77 | 7.44 | 0.269 |
| Right putamen | 5.81 | 0.37 | 5.25 | 6.58 | 5.50 | 0.60 | 4.49 | 6.36 | 0.360 |
| Left caudate nucleus | 4.07 | 0.51 | 3.25 | 4.96 | 3.90 | 0.46 | 3.13 | 4.79 | 0.628 |
| Right caudate nucleus | 4.14 | 0.38 | 3.51 | 4.73 | 3.74 | 0.40 | 3.15 | 4.58 | 0.063 |
| Left pallidum | 1.87 | 0.34 | 1.49 | 2.74 | 1.77 | 0.22 | 1.29 | 2.09 | 0.895 |
| Right pallidum | 2.11 | 0.20 | 1.86 | 2.45 | 1.89 | 0.32 | 1.39 | 2.42 | 0.149 |
| Left thalamus | 8.38 | 0.95 | 7.10 | 10.23 | 8.13 | 0.73 | 6.84 | 9.29 | 0.779 |
| Right thalamus | 7.43 | 0.67 | 6.50 | 8.65 | 7.04 | 0.59 | 6.14 | 8.08 | 0.509 |
| **Corpus callosum*** |  |  |  |  |  |  |  |  |  |
| Corpus callosum posterior | 1.01 | 0.17 | 0.73 | 1.27 | 0.87 | 0.12 | 0.70 | 1.09 | 0.097 |
| Corpus callosum mid-posterior | 0.47 | 0.08 | 0.31 | 0.59 | 0.39 | 0.07 | 0.25 | 0.54 | 0.060 |
| Corpus callosum central | 0.46 | 0.08 | 0.32 | 0.64 | 0.41 | 0.10 | 0.25 | 0.61 | 0.345 |
| Corpus callosum mid-anterior | 0.48 | 0.06 | 0.35 | 0.56 | 0.44 | 0.08 | 0.28 | 0.61 | 0.396 |
| Corpus callosum anterior | 0.93 | 0.18 | 0.70 | 1.36 | 0.80 | 0.11 | 0.56 | 0.96 | 0.147 |
| Corpus callosum total | 3.35 | 0.48 | 2.61 | 4.28 | 2.92 | 0.37 | 2.06 | 3.44 | 0.079 |
| **Cerebellum** |  |  |  |  |  |  |  |  |  |
| Left cerebellar cortical | 51.77 | 4.70 | 43.47 | 60.15 | 51.82 | 3.84 | 45.61 | 57.73 | 0.135 |
| Right cerebellar cortical | 53.52 | 5.74 | 42.16 | 61.40 | 54.12 | 3.78 | 45.64 | 58.77 | 0.098 |
| Left cerebellar white matter | 13.72 | 1.45 | 11.52 | 15.87 | 15.30 | 2.01 | 13.12 | 19.55 | 0.012 |
| Right cerebellar white matter | 14.24 | 1.62 | 11.37 | 17.36 | 15.46 | 2.30 | 12.95 | 21.02 | 0.035 |

*, corpus callosum volume was computed based on a mid-sagittal slice of 5 mm thickness.
